# Supplementary material for: Polyclonal antibody responses to HIV Env immunogens resolved using cryoEM
Source: Nat Commun. 2021 Aug 10;12:4817. doi: 10.1038/s41467-021-25087-4 (PMC8355326; doi:10.1038/s41467-021-25087-4)
Supplement: Supplementary file 1 — Supplementary Information [file 41467_2021_25087_MOESM1_ESM.pdf]

Supplementary Information

Polyclonal antibody responses to HIV Env immunogens resolved using cryoEM

Aleksandar Antanasijevic[1,2], Leigh M. Sewall[1,2], Christopher A. Cottrell[1,2], Diane G. Carnathan[3], Luis E. Jimenez[3], Julia T. Ngo[3], Jennifer B. Silverman[3], Bettina Groschel[4], Erik Georgeson[4], Jinal Bhiman[4], Raiza Bastidas[4], Celia LaBranche[5], Joel D. Allen[6], Jeffrey Copps[1], Hailee R. Perrett[1], Kimmo Rantalainen[4], Fabien Cannac[1], Yuhe R. Yang[1], Alba Torrents de la Peña[1], Rebeca Froes Rocha[1], Zachary T. Berndsen[1], David Baker[7,8], Neil P. King[7], Rogier W. Sanders[9,10], John P. Moore[10], Shane Crotty[11], Max Crispin[6], David C. Montefiori[5], Dennis R. Burton[2,4], William R. Schief[2,4], Guido Silvestri[3], Andrew B. Ward[1,2#]

Author affiliations:

1- Department of Integrative, Structural and Computational Biology, The Scripps Research Institute, La Jolla, CA 92037, USA.
2- International AIDS Vaccine Initiative Neutralizing Antibody Center, the Collaboration for AIDS Vaccine Discovery (CAVD) and Scripps Consortium for HIV/AIDS Vaccine Development (CHAVD), The Scripps Research Institute, La Jolla, CA 92037, USA.
3- Division of Microbiology and Immunology, Yerkes National Primate Research Center, Emory University, Atlanta, GA 30329, USA.
4- Department of Immunology and Microbiology, The Scripps Research Institute, La Jolla, CA 92037, USA.
5- Department of Surgery, Duke University Medical Center, Durham, NC, 27710, USA.
6- School of Biological Sciences, University of Southampton, Southampton, SO17 1BJ, United Kingdom.
7- Institute for Protein Design, Department of Biochemistry, University of Washington, Seattle, WA 98195, USA.
8- Howard Hughes Medical Institute, Chevy Chase, MD 20815, USA.
9- Academic Medical Center (AMC), University of Amsterdam, Amsterdam 1105AZ, Netherlands.
10- Weill Cornell Medicine, Cornell University, New York, NY 10065, USA.
11- La Jolla Institute for Immunology, La Jolla, CA 92037, USA.

# Correspondence should be addressed to: Andrew B. Ward, andrew@scripps.edu

Supplementary Table 1. Anti-trimer binding antibody titers (midpoint, EC_{50}) determined via ELISA for plasma samples collected at indicated time points. Color coding: white - value not determined (N/D) or material not available (N/A); light-gray - no detectable binding (EC_{50} < 20); light orange - 20 < EC_{50} < 100); medium orange - 100 < EC_{50} < 500; dark orange - 500 < EC_{50} < 1000; brown - EC_{50} > 1000.

**Group / Immunogen**	Animal ID	**Week 6**	**Week 10**	**Week 26**	**Week 38**
**Grp 1** BG505 SOSIP MD39	32034	< 20	93	167	164
	32113	< 20	196	479	1233
	34943	< 20	75	166	101
	34909	< 20	115	109	N/D
	33104	< 20	151	323	231
	33395	< 20	288	516	253
**Grp 2** BG505 SOSIP.v5.2 N241/N289	34686	61	277	134	86
	33182	36	1073	587	364
	33311	35	1921	121	626
	CD99	54	595	242	325
	33203	47	381	310	233
	CG41	42	1430	256	378
**Grp 3** BG505 SOSIP.v5.2(7S) N241/N289 T33-31 NP	33172	33	858	431	595
	34919	< 20	342	384	356
	34167	64	929	860	832
	33065	30	211	178	295
	33176#	54	451	N/A	N/A
	34725	57	480	397	850

This animal was euthanized early due to chronic health issues. Sera samples were not available for Week 26 and Week 38 time points (see Methods section for details).

N/D – Not determined

N/A – Samples not available

Supplementary Table 2. Autologous neutralization titers (ID_{50}) against BG505-based pseudovirus. Midpoint neutralization titers (ID_{50}) were determined using sera samples collected at different time points (indicated in the top row). Neutralization assays with VRC01 were also performed (positive control). Color coding: light gray - no neutralization (ID_{50} < 40); light orange - very weak neutralization (40 < ID_{50} < 100); medium orange - moderate neutralization (100 < ID_{50} < 500); dark orange - strong neutralization (500 < ID_{50} < 2000); brown - very strong neutralization (ID_{50} > 2000).

		Week 10	Week 26	Week 38
	Virus	BG505	BG505	BG505
Group / Immunogen	Animal ID			
**Grp 1** BG505 SOSIP MD39	32034	41	322	1131
	32113	< 40	< 40	< 40
	34943	< 40	< 40	< 40
	34909	84	506	1212
	33104	< 40	< 40	< 40
	33395	42	267	1477
**Grp 2** BG505 SOSIP.v5.2 N241/N289	34686	< 40	< 40	< 40
	33182	< 40	< 40	< 40
	33311	< 40	604	8599
	CD99	< 40	< 40	111
	33203	< 40	66	123
	CG41	< 40	378	507
**Grp 3** BG505 SOSIP.v5.2(7S) N241/N289 T33-31 NP	33172	< 40	< 40	407
	34919	< 40	< 40	< 40
	34167	< 40	< 40	< 40
	33065	< 40	47	232
	33176#	< 40	N/A	N/A
	34725	< 40	< 40	< 40
VRC01	IC_{50} (µg/ml)	0.05	0.04	0.05

This animal was euthanized early in the study due to chronic health issues. Sera samples were not available for Week 26 and Week 38 time points (see Methods section for details)
N/A – Samples not available

Supplementary Table 3. Cryo-EM data collection information

	Rh.32034 Poly. Fab + BG505 SOSIP MD39	Rh.33104 Poly. Fab + BG505 SOSIP MD39	Rh.33311 Poly. Fab + BG505 SOSIP v5.2 N241/N289	Rh.33172 Poly. Fab + BG505 SOSIP v5.2(7S) N241/N289	BG505 SOSIP T33-31-NP
**Microscope**	Titan Krios	Titan Krios	Titan Krios	Titan Krios	Talos Arctica
**Voltage (kV)**	300	300	300	300	200
**Detector**	Gatan K2 Summit	Gatan K2 Summit	Gatan K2 Summit	Gatan K2 Summit	Gatan K2 Summit
**Recording mode**	Counting	Counting	Counting	Counting	Counting
**Magnification**	29,000	29,000	29,000	29,000	36,000
**Movie micrograph pixel size**	1.03	1.03	1.03	1.03	1.15
**Dose rate (e^{-}/Å^2/s)**	5.98	4.55	4.55	5.63	4.88
**No. of frames per movie micrograph**	30	39	39	36	41
**Frame exposure time (ms)**	250	250	250	250	250
**Movie micrograph exposure time (s)**	7.50	9.75	9.75	9.00	10.25
**Total dose (e^{-}/Å^2)**	44.9	44.3	44.3	50.7	50.0
**Grid Type**	UltrAuFoil R 1.2/1.3	UltrAuFoil R 1.2/1.3	UltrAuFoil R 1.2/1.3	Quantifoil R 2/1	Quantifoil R 2/1
**Under focus range (μm)**	0.6 – 1.6	0.6 – 1.6	0.6 – 1.6	0.6 – 1.6	0.8 – 2.0
**Number of movie micrographs**	3,194	3,580	6,268	4,521	1,751

Supplementary Table 4. Map and model refinement information

Animal ID	Rh.32034 (Grp 1)				Rh.33104 (Grp 1)				Rh.33311 (Grp 2)							
**Complex ID**	**pAbC-1**	**pAbC-2**	**pAbC-3**	**pAbC-4**	**pAbC-1**	**pAbC-2**	**pAbC-3**	**pAbC-4**	**pAbC-1**	**pAbC-2**	**pAbC-3**	**pAbC-4**	**pAbC-5**	**pAbC-6**	**pAbC-7**	**pAbC-8**
**Antigen**	**BG505 SOSIP MD39**				**BG505 SOSIP MD39**				**BG505 SOSIP.v5.2 N241/N289**							
**Number of picked particles**	591,593				502,068				1,267,693							
**Particles after 2D classification**	327,148				230,034				503,502							
**Particles after symmetry expansion**	981,444				690,102				1,510,506							
**Particles in the final map**	48,919	37,870	47,950	106,016	81,561	27,719	58,378	11,534	82,126	28,188	39,028	27,483	52,082	12,653	70,307	29,622
**Map symmetry**	C1	C1	C1	C1	C1	C1	C1	C1	C1	C1	C1	C1	C1	C1	C1	C1
**Map sharpening B-factor**	-79.6	-94.3	-7.1	-12.1	-87.6	-90.8	-83.4	-93.9	-103.8	-118.7	-122.1	-103.6	-126.5	-131.0	-121.8	-117.2
**Map Resolution**	3.4	3.6	3.6	3.8	3.3	3.7	3.4	4.6	3.7	4.5	4.1	4	4.2	6.6	3.8	4.5
**EMDB ID**	23223	23224	23225	23226	23227	23228	23229	23230	23236	23237	23238	23239	23240	23241	23242	23243
**Residues**	2010	2004	1992	1988	2007	2003	2022	1972	2082	1978	2012	2039	2031	N/A	2026	1976
**Amino acids**	1910	1901	1898	1888	1910	1909	1911	1871	1994	1893	1926	1936	1951	N/A	1930	1897
**Carbohydrates**	100	103	94	100	97	94	111	101	88	85	86	103	80	N/A	96	79
**RMSD Bonds (4σ)**	0.022	0.021	0.021	0.022	0.023	0.021	0.025	0.021	0.021	0.021	0.02	0.021	0.02	N/A	0.022	0.02
**RMSD Angles (4σ)**	1.653	1.722	1.684	1.723	1.709	1.687	1.698	1.669	1.674	1.702	1.679	1.647	1.633	N/A	1.716	1.71
**Ramachandran**																
**Outliers (%)**	0	0	0	0	0	0	0	0	0	0	0	0	0	N/A	0	0
**Allowed (%)**	1.88	3.5	3.19	2.61	2.09	2.57	2.41	2.25	2.19	3.35	2.01	2.53	2.66	N/A	3.59	2.86
**Favored (%)**	98.12	96.5	96.81	97.39	97.91	97.43	97.59	97.75	97.81	96.65	97.99	97.47	97.34	N/A	96.41	97.14
**Rotamer outliers (%)**	0	0	0	0	0	0	0	0	0	0	0	0	0	N/A	0	0
**Clash score**	1.22	1.99	2.17	2.07	1.19	2.16	0.9	2.6	1.45	2.67	1.34	0.91	1.87	N/A	1.01	2.97
**Molprobity score**	0.84	1.2	1.19	1.1	0.85	1.1	1.11	1.1	0.93	1.27	0.87	0.88	1.08	N/A	1.04	1.24
**EMRinger score**	4.17	3.36	3.75	3.71	4.33	3.73	4.33	0.77	3.25	0.93	2.29	2.79	2.39	N/A	2.99	1.38
**PDB ID**	7L86	7L87	7L88	7L89	7L8A	7L8B	7L8C	7L8D	7L8T	7L8U	7L8W	7L8X	7L8Y	N/A	7L8Z	7L90

Supplementary Table 4. Map and model refinement information (continued)

Animal ID	N/A			Rh.33172 (Grp 3)				
**Complex ID**	**T33-31 NP core**	**BG505 SOSIP Comp A**	**BG505 SOSIP Comp B**	**pAbC-1**	**pAbC-2**	**pAbC-3**	**pAbC-4**	**pAbC-5**
**Antigen**	N/A			**BG505 SOSIP.v5.2(7S) N241/N289**				
**Number of picked particles**	223,099	441,476*	441,476*	1,121,581				
**Particles after 2D classification**	214,224	353,064	316,582	456,268				
**Particles after symmetry expansion**	N/A			1,368,804				
**Particles in the final map**	110,369	106,478	64,726	31,914	66,171	33,361	28,929	33,802
**Map symmetry**	T	C3	C3	C1	C1	C1	C1	C1
**Map sharpening B-factor**	-53.1	-70.5	-164.5	-124.0	-105.9	-95.1	-112.4	-120.8
**Map Resolution**	2.9	3.7	3.8	4.2	3.7	4.1	4.3	4.0
**EMDB ID**	23222	23218	23219	23231	23232	23233	23235	23234
**Residues**	2664	1872	1857	2007	2027	2002	2029	N/A
**Amino acids**	2664	1770	1761	1912	1939	1904	1925	N/A
**Carbohydrates**	0	102	96	95	88	98	104	N/A
**RMSD Bonds (4σ)**	0.025	0.023	0.022	0.021	0.021	0.021	0.021	N/A
**RMSD Angles (4σ)**	1.653	1.701	1.645	1.691	1.654	1.701	1.687	N/A
**Ramachandran**								
**Outliers (%)**	0	0	0	0	0	0	0	N/A
**Allowed (%)**	2.29	1.21	1.39	1.76	2	2.15	1.91	N/A
**Favored (%)**	97.71	98.79	98.61	98.24	98	97.85	98.09	N/A
**Rotamer outliers (%)**	0	0	0	0	0	0	0	N/A
**Clash score**	2.46	0.2	1.76	2.15	1.34	1.06	1.48	N/A
**Molprobity score**	1.1	0.58	0.93	0.99	0.86	0.84	0.89	N/A
**EMRinger score**	4.84	2.9	2.40	2.19	3.31	1.87	1.9	N/A
**PDB ID**	7L85	7L7T	7L7U	7L8E	7L8F	7L8G	7L8S	N/A

Supplementary Table 5. Neutralization titers (ID_{50}) against mutant BG505 pseudoviruses. Midpoint neutralization titers (ID_{50}) were determined using sera samples collected at the week 38 time point. Neutralization assays with MLV (negative control) and CH01-31 (positive control and reference) were performed. Color coding: light gray - no neutralization ($ID_{50} < 20$); light orange - very weak neutralization ($20 < ID_{50} < 100$); medium orange - moderate neutralization ($100 < ID_{50} < 500$); dark orange - strong neutralization ($500 < ID_{50} < 2000$); brown - very strong neutralization ($ID_{50} > 2000$).

		Week 38						
	Virus	SVA **MLV**	BG505 **T332N**	BG505 **T332N.T465N**	BG505 **T332N.S241N**	BG505 **T332N.P291T**	BG505 **T332N.N156A**	BG505 **T332N.N611A**
Group / Immunogen	Animal ID							
**Grp 1** BG505 SOSIP MD39	32034	<20	714	<20	870	413	417	N/D
	32113	<20	<20	<20	<20	<20	<20	N/D
	34943	<20	36	<20	<20	<20	<20	N/D
	34909	<20	285	<20	314	126	<20	N/D
	33104	<20	<20	<20	<20	<20	<20	N/D
	33395	<20	1129	338	1137	749	627	N/D
**Grp 2** BG505 SOSIP.v5.2 N241/N289	34686	<20	<20	<20	N/D	<20	N/D	25
	33182	<20	<20	<20	N/D	<20	N/D	72
	33311	<20	3073	200	N/D	1383	N/D	4237
	CD99	<20	67	41	N/D	34	N/D	60
	33203	<20	27	<20	N/D	<20	N/D	41
	CG41	<20	1608	<20	N/D	759	N/D	1595
**Grp 3** BG505 SOSIP.v5.2(7S) N241/N289 T33-31 NP	33172	<20	229	230	207	177	320	N/D
	34919	<20	<20	<20	<20	<20	<20	N/D
	34167	<20	<20	<20	<20	<20	107	N/D
	33065	<20	171	142	172	110	299	N/D
	33176#	N/A	N/A	N/A	N/A	N/A	N/A	N/A
	34725	<20	<20	<20	<20	<20	370	N/D
CH01-31	IC_{50} (µg/ml)	>25	0.03	0.028	0.028	0.013	0.036	0.019

#This animal was euthanized early in the study due to chronic health issues. Sera samples were not available for Week 26 and Week 38 time points (see Methods section for details)

N/D – Not determined

N/A – Samples not available

Supplementary Table 6. Site-specific glycosylation analysis of BG505-SOSIP-presenting nanoparticle components and free BG505-SOSIPv5.2(7S) N241/N289. Glycan compositions corresponding to oligomannose/hybrid-type (green) and fully processed complex type (magenta) are shown at each potential N-linked glycosylation site (PNGS). Unglycosylated PNGS are shown in grey. Oligomannose-type glycans are divided with respect to the number of mannose residues present while hybrid-type glycans are categorized according to the presence/absence of fucose. For complex-type glycans, the categorization was performed according to the number of processed antenna and the presence of fucose. For PNGS where only low intensity peptides were present, the categorization was not performed and so in the table they are merged to cover all possible oligomannose/hybrid compositions or complex-type glycans. The unliganded trimer data is adapted from a manuscript by Antanasijevic et al., 2020; and displayed here as a reference for comparison.

BG505 SOSIP.v5.2(7S) N241/N289

BG505 SOSIP.v5.2(7S) N241/N289	N88	N133	N137	N156	N160	N185e	N185h	N197	N234	N241	N262	N276	N289	N295	N301	N332	N339	N355	N363	N386	N392	N398	N406	N411	N448	N462	N611	N618	N625	N637
High Mannose	21	60	40	88	100	6	0	66	84	85	100	67	81	98	81	100	64	22	100	n.d.	n.d.	n.d.	0	100	100	1	1	0	24	34
M9	0				1	0		10				0				29	37	0	63						50	0		0		0
M8	0				21	2		11				5				26	21	1	28						31	0		0		0
M7	0				26	1		14				5				31	4	1	5						7	0		0		9
M6	6				21	0		6				0				2	0	2	2						3	0		0		0
M5	13				24	3		17				6				10	2	8	1						6	0		0		6
M4	0				7	0		0				2				1	1	2	0						2	0		0		0
M3	0				0	0		0				0				0	0	1	0						0	0		0		0
Hybrid	2				0	0		7				37				0	0	2	0						0	0		0		8
FHybrid	0				0	0		0				12				0	0	6	0						0	0		0		11
A1	10				0	0		0				0				0	0	1	0						0	0		0		0
FA1	0				0	2		0				2				0	0	11	0						0	2		0		0
A2/A1B	28				0	0		0				13				0	0	0	0						0	0		0		0
FA2/FA1B	11	11	15	1	0	58	100	27	1	2	0	9	0	2	0	0	0	32	0	n.d.	n.d.	n.d.	100	0	0	45	50	26	1	13
A3/A2B	13				0	0		0				4				0	0	0	0						0	0		0		0
FA3/FA2B	17				0	15		0				5				0	0	29	0						0	50		65		0
A4/A3B	0				0	0		0				0				0	0	0	0						0	0		0		0
FA4/FA3B	0				0	0		0				0				0	0	1	0						0	2		9		0
Unoccupied	0	30	44	11	0	19	0	0	15	13	0	0	19	0	19	0	36	0	0	n.d.	n.d.	n.d.	0	0	0	0	49	0	75	44

BG505 SOSIP-T33-31A

BG505 SOSIP-T33-31A	N88	N133	N137	N156	N160	N185e	N185h	N197	N234	N241	N262	N276	N289	N295	N301	N332	N339	N355	N363	N386	N392	N398	N406	N411	N448	N462	N611	N618	N625	N637
High Mannose	87	87	57	100	100	18	3	100	100	99	100	98	73	97	93	100	100	85	100	100	100	74	64	94	100	6	0	77	71	26
M9	0				11	0	0	54				0				81	64	0	62						69	0	0			2
M8	0				36	1	0	32				41				16	25	11	32						22	0	0			8
M7	6				23	2	0	15				25				2	6	17	4						5	1	0			5
M6	16				17	3	0	0				8				0	3	11	2						2	1	0			2
M5	56				14	10	3	0				6				0	2	37	0						1	3	0			0
M4	4				0	0	0	0				2				0	0	2	0						0	0	0			0
M3	0				0	0	0	0				0				0	0	0	0						0	0	0			0
Hybrid	5				0	1	0	0				15				0	0	4	0						0	0	0			4
FHybrid	0				0	0	0	0				0				0	0	3	0						0	1	0			4
A1	8				0	1	0	0				0				0	0	1	0						0	3	0			0
FA1	0				0	3	2	0				0				0	0	3	0						0	4	0			1
A2/A1B	4				0	0	0	0				1				0	0	0	0						0	2	0			0
FA2/FA1B	0	0	11	0	0	36	77	0	0	0	0	1	0	0	0	0	0	4	0	0	0	26	36	6	0	53	0	21	29	45
A3/A2B	1				0	0	0	0				0				0	0	0	0						0	1	0			0
FA3/FA2B	0				0	15	18	0				0				0	0	4	0						0	28	90			27
A4/A3B	0				0	0	0	0				0				0	0	0	0						0	0	0			0
FA4/FA3B	0				0	0	0	0				0				0	0	0	0						0	2	10			0
Unoccupied	0	13	32	0	0	26	0	0	0	1	0	0	26	3	7	0	0	0	0	0	0	0	0	0	0	0	0	2	0	0

BG505 SOSIP-T33-31B

BG505 SOSIP-T33-31B	N88	N133	N137	N156	N160	N185e	N185h	N197	N234	N241	N262	N276	N289	N295	N301	N332	N339	N355	N363	N386	N392	N398	N406	N411	N448	N462	N611	N618	N625	N637
High Mannose	55	92	54	100	100	9	3	60	100	100	100	89	69	91	79	100	76	79	100	100	100	69	57	98	100	3	26	31	78	57
M9	0				3	0	0	8				0				66	58	0	74	100					57	0				0
M8	2				0	1	0	8				21				25	17	7	21	0					29	0				6
M7	4				29	1	0	6				19				4	0	11	4	0					7	0				12
M6	16				28	1	0	5				10				2	1	11	1	0					4	0				8
M5	18				37	5	3	13				9				3	0	36	0	0					2	2				5
M4	2				3	0	0	2				4				1	0	2	0	0					1	0				0
M3	5				0	0	0	0				1				0	0	0	0	0					0	0				0
Hybrid	7				0	0	0	11				24				0	0	6	0	0					0	0				13
FHybrid	1				0	0	0	6				0				0	0	6	0	0					0	0				13
A1	8				0	1	0	1				3				0	0	1	0	0					0	1				1
FA1	1				0	4	2	7				1				0	0	6	0	0					0	4				13
A2/A1B	29				0	0	0	1				5				0	0	0	0	0					0	14				0
FA2/FA1B	0	0	8	0	0	46	83	26	0	0	0	1	0	2	1	0	0	7	0	0	0	29	42	0	0	50	66	68	0	29
A3/A2B	0				0	0	0	0				2				0	0	0	0	0					0	3				0
FA3/FA2B	1				0	17	12	5				0				0	0	5	0	0					0	24				0
A4/A3B	0				0	0	0	0				0				0	0	0	0	0					0	0				0
FA4/FA3B	0				0	0	0	0				0				0	0	0	0	0					0	1				0
Unoccupied	5	7	39	0	0	22	0	0	0	0	0	0	31	7	20	0	24	0	0	0	0	2	2	2	0	0	8	0	22	0

Supplementary Table 7. Nucleotide sequences of the constructs used in this study

BG505 SOSIP.v5.2(7S) N241/N289	ATGAAAAGGGGGCTGTGCTGTGTGCTGCTGCTGTGCGGGGCTGTGTTTGTGTCACCCAGTCAGGAAATCCACGCCAGATTCCG<br>GAGAGGAGCTAGGGCAGAAAACCTGTGGGTGACAGTCTACTATGGCGTGCCTGTCTGGAAGGACGCCGAGACCACACTGTTTT<br>GCGCTTCCGATGCCAAGGCTTACGAAACTAAGAAACACAATGTGTGGGCTACCCATTGCTGTGTCCCAACAGACCCAAACCCC<br>CAGGAAATCCACCTGGAGAATGTGACCGAGGAATTCAACATGTGGAAGAACAATATGGTGGAGCAGATGCATACAGACATCA<br>TTTCCCTGTGGGATCAGTCTCTGAAGCCTTGCGTGAAACTGACCCCACTGTGCGTCACACTCCAGTGTACAAACGTGACTAACA<br>ATATCACCGACGATATGCGCGGAGAACTGAAGAATTGTTCTTTCAACATGACTACCGAGCTGAGGGACAAGAAACAGAAAGT<br>GTACAGTCTGTTTTATCGCCTGGATGTGGTCCAGATCAATGAAAACCAGGGGAATAGAAGTAACAATTCAAACAAGGAGTACA<br>GGCTGATCAATTGCAACACCAGTGCCATTACACAGGCTTGTCCAAAAGTGTCATTTGAACCTATCCCAATTCATTATTGCGCAC<br>CTGCCGGCTTCGCCATCCTGAAGTGTAAAGATAAGAAGTTCAACGGCACTGGGCCCTGCACTAATGTGAGCACTGTCCAGTGT<br>ACCCACGGGATTAAGCCTGTGGTCTCCACCCAGCTGCTGCTGAATGGATCTCTGGCCGAGGAAGAAGTGATCATCCGGTCTGA<br>GAACATCACTAACAACGCTAAGAACATCCTGGTGCAGTTAAACGAATCCGTCCAGATTAATTGCACTAGACCTAACAATAACA<br>CCGTGAAATCTATCCGCATTGGACCCGGCCAGTGGTTTTATTATACCGGCGACATCATTGGGGATATCCGGCAGGCACACTGTA<br>ATGTGAGCAAGGCTACATGGAACGAGACTCTGGGGAAGGTGGTCAAACAGCTGCGCAAACATTTCGGAAATAACACCATCAT<br>TCGATTTGCCAATAGCTCCGGCGGGGACCTGGAAGTGACAACTCACAGCTTCAACTGCGGAGGCGAGTTCTTTTACTGTAACA<br>CAAGTGGCCTGTTTAATTCAACTTGGATCAGCAACACCTCCGTGCAGGGCTCTAATTCTACCGGCTCTAACGATAGTATCACAC<br>TGCCATGCCGGATTAAGCAGATCATTAATATGTGGCAGAGAATCGGGCAGGCAATGTATGCCCCCCCTATCCAGGGAGTGATT<br>CGATGTGTCAGCAATATCACAGGCCTGATTCTGACTAGAGACGGGGGATCAACAAACAGCACCACAGAGACTTTCAGGCCCGG<br>CGGGGGAGACATGCGAGATAACTGGCGGTCCGAACTGTACAAGTATAAAGTGGTCAAGATCGAGCCACTGGGAGTGGCACCA<br>ACCCGATGCAAAAGGCGAGTGGTCGGACGACGAAGAAGGCGACGAGCTGTGGGGATTGGAGCAGTCTCCCTGGGCTTTCTGG<br>GGGCCGCTGGATCTACAATGGGCGCAGCCAGTATGACTCTGACCGTCCAGGCCAGGAATCTGCTGTCAGGGATCGTGCAGCAG<br>CAGAGCAACCTGCTGCGCGCTCCTGAATGCCAGCAGCATCTGCTGAAGGACACCCACTGGGGCATCAAGCAGCTCCAGGCACG<br>AGTGCTGGCAGTCGAGCACTACCTGAGAGATCAGCAGCTGCTGGGAATCTGGGGGTGCAGCGGAAAGCTGATTTGCTGTACCA<br>ATGTGCCTTGGAACTCTAGTTGGAGCAATAGAAACCTGTCCGAAATCTGGGACAATATGACATGGCTCCAGTGGGATAAGGAG<br>ATTAGCAACTACACTCAGATCATCTACGGCCTGCTGGAAGAGTCCCAGAATCAGCAGGAGAAGAACGAGCAGGACCTGCTGG<br>AGCTGGAC
BG505 SOSIP.v5.2 N241/N289	ATGGACGCTATGAAAAGGGGGCTGTGCTGTGTGCTGCTGCTGTGCGGGGCTGTGTTTGTGTCACCCAGTCAGGAAATCCACGC<br>CAGATTCCGGAGAGGAGCTAGGGCAGAAAACCTGTGGGTGACAGTCTACTATGGCGTGCCTGTCTGGAAGGACGCCGAGACC<br>ACACTGTTTTGCGCTTCCGATGCCAAGGCTTACGAAACTAAGAAACACAATGTGTGGGCTACCCATTGCTGTGTCCCAACAGAC<br>CCAAACCCCCAGGAAATCCACCTGGAGAATGTGACCGAGGAATTCAACATGTGGAAGAACAATATGGTGGAGCAGATGCATA<br>CAGACATCATTTCCCTGTGGGATCAGTCTCTGAAGCCTTGCGTGAAACTGACCCCACTGTGCGTCACACTCCAGTGTACAAACG<br>TGACTAACAATATCACCGACGATATGCGCGGAGAACTGAAGAATTGTTCTTTCAACATGACTACCGAGCTGAGGGACAAGAAA<br>CAGAAAGTGTACAGTCTGTTTTATCGCCTGGATGTGGTCCAGATCAATGAAAACCAGGGGAATAGAAGTAACAATTCAAACAA<br>GGAGTACAGGCTGATCAATTGCAACACCAGTGCCATTACACAGGCTTGTCCAAAAGTGTCATTTGAACCTATCCCAATTCATTA<br>TTGCGCACCTGCCGGCTTCGCCATCCTGAAGTGTAAAGATAAGAAGTTCAACGGCACTGGGCCCTGCactaacGTGAGCACTGTCC<br>AGTGTACCCACGGGATTAAGCCTGTGGTCTCCACCCAGCTGCTGCTGAATGGATCTCTGGCCGAGGAAGAAGTGATCATCCGG<br>TCTGAGAACATCACTAACAACGCTAAGAACATCCTGGTGCAGCTGAACGAGTCCGTCCAGATTAATTGCACTAGACCTAACAA<br>TAACACCAGGAAATCTATCCGCATTGGACCCGGCCAGTGGTTTTATGCTACCGGCGACATCATTGGGGATATCCGGCAGGCAC<br>ACTGTAATGTGAGCAAGGCTACATGGAACGAGACTCTGGGGAAGGTGGTCAAACAGCTGCGCAAACATTTCGGAAATAACAC<br>CATCATTCGATTTGCCAATAGCTCCGGCGGGGACCTGGAAGTGACAACTCACAGCTTCAACTGCGGAGGCGAGTTCTTTTACTG<br>TAACACAAGTGGCCTGTTTAATTCAACTTGGATCAGCAACACCTCCGTGCAGGGCTCTAATTCTACCGGCTCTAACGATAGTAT<br>CACACTGCCATGCCGGATTAAGCAGATCATTAATATGTGGCAGAGAATCGGGCAGGCAATGTATGCCCCCCCTATCCAGGGAG<br>TGATTCGATGTGTCAGCAATATCACAGGCCTGATTCTGACTAGAGACGGGGGATCAACAAACAGCACCACAGAGACTTTCAGG<br>CCCGGCGGGGGAGACATGCGAGATAACTGGCGGTCCGAACTGTACAAGTATAAAGTGGTCAAGATCGAGCCACTGGGAGTGG<br>CACCAACCCGATGTAAAAGGCGAGTGGTCGGACGACGAAGAAGGCGACGAGCTGTGGGGATTGGAGCAGTCTTCCTGGGCTT<br>TCTGGGGGCCGCTGGATCTACAATGGGCGCAGCCAGTATGACTCTGACCGTCCAGGCCAGGAATCTGCTGTCAGGGATCGTGC<br>AGCAGCAGAGCAACCTGCTGCGCGCTCCCGAATGCCAGCAGCATCTGCTGAAGCTGACCGTGTGGGGCATCAAGCAGCTCCAG<br>GCACGAGTGCTGGCAGTCGAGCGGTACCTGAGAGATCAGCAGCTGCTGGGAATCTGGGGGTGCAGCGGAAAGCTGATTTGCT<br>GTACCAATGTGCCTTGGaacTCTAcTTGGAGCAATAGAAACCTGTCCGAAATCTGGGACAATATGACATGGCTCCAGTGGGATAA<br>GGAGATTAGCAACTACACTCAGATCATCTACGGCCTGCTGGAAGAGTCCCAGAATCAGCAGGAGAAGAACGAGCAGGACCTG<br>CTGGCCCTGGATTAA
BG505 SOSIP MD39	ATGAAAAGAGGCCTGTGCTGTGTGCTGCTGCTGTGTGGCGCCGTGTTCGTGAGCCCCTCTCAGGAGATCCACGCCAGATTCAG<br>GCGAGGCGCCCGGGCCGAGAACCTGTGGGTGACCGTGTACTACGGCGTTCCAGTGTGGAAAGACGCCGAGACAACCCTGTTCT<br>GCGCCAGCGACGCCAAGGCCTACGAGACCGAAAAGCACAACGTGTGGGCCACACATGCCTGCGTGCCCACCGACCCCAACCC<br>CCAGGAGATCCACCTGGAAAACGTGACCGAGGAATTCAACATGTGGAAGAACAACATGGTGGAACAGATGCACGAGGACATA<br>ATCTCCCTGTGGGACCAGTCTCTGAAACCTTGCGTGAAGCTGACACCCCTGTGCGTTACACTGCAGTGTACCAATGTGACCAAT<br>AATATCACCGACGACATGAGAGGCGAGCTGAAAAACTGCAGCTTTAACATGACCACAGAACTGAGAGACAAGAAGCAGAAGG<br>TGTACAGCCTGTTTTATAGACTGGACGTGGTGCAGATCAACGAGAACCAGGGCAACAGATCCAACAACAGCAACAAGGAGTA<br>CAGACTGATTAACTGCAATACAAGCGCCATCACCCAAGCTTGTCCTAAGGTGAGCTTCGAGCCTATCCCCATCCACTACTGTGC<br>CCCTGCCGGCTTCGCCATCCTGAAGTGCAAAGACAAGAAATTCAACGGCACCGGCCCTTGCCCTAGCGTGAGCACCGTTCAGT<br>GCACCCATGGCATCAAGCCTGTGGTCAGCACCCAGCTGCTGCTCAACGGCTCTCTGGCCGAAGAAGAGGTGATCATTCGGAGC<br>GAGAACATCACCAACAACGCCAAGAATATTCTGGTGCAACTGAACACCCCAGTGCAGATCAACTGTACAAGACCTAACAACA<br>ACACCGTGAAATCCATCAGAATTGGGCCCGGCCAGGCCTTCTACTACACCGGCGACATCATCGGAGATATCAGACAGGCCCAC<br>TGCAACGTGTCCAAGGCCACATGGAACGAGACACTGGGAAAGGTGGTGAAACAGCTGAGAAAGCACTTCGGCAACAACACCA<br>TCATCCGTTTCGCCCAGAGCAGCGGCGGCGACCTGGAAGTGACCACACACAGCTTCAACTGCGGCGGCGAGTTCTTCTATTGC<br>AACACCAGCGGACTGTTTAACAGCACCTGGATCAGCAACACCTCCGTGCAGGGAAGCAATTCTACAGGCAGCAATGATAGCAT<br>CACTCTGCCTTGTAGAATCAAGCAGATCATTAACATGTGGCAGCGGATCGGCCAGGCAATGTACGCCCCACCTATCCAGGGCG<br>TGATCCGGTGCGTGAGCAACATCACAGGCCTGATCCTGACCCGGGACGGCGGAAGCACAAACTCCACAACCGAAACCTTTAGA<br>CCTGGCGGCGGCGATATGCGGGACAACTGGCGGAGCGAGCTGTACAAGTACAAGGTGGTGAAGATCGAGCCTCTGGGCGTCG<br>CCCCTACAAGATGCAAGAGACGGGTGGTCGGAAGACGGCGCCGGCGGAGAGCTGTGGGCATCGGCGCCGTCAGCCTGGGATT<br>CCTGGGCGCCGCTGGTTCTACCATGGGCGCTGCTTCTATGACTCTGACCGTGCAGGCTAGAAATCTGCTGTCTGGGATCGTGCA<br>GCAGCAGAGCAACCTTCTGAGAGCCCCAGAACCTCAGCAACACCTGCTGAAGGACACCCACTGGGGCATCAAGCAACTGCAG<br>GCCAGAGTGCTGGCAGTTGAGCACTACCTGAGAGATCAGCAACTGCTGGGAATCTGGGGATGTAGCGGCAAGCTGATCTGCTG<br>CACCAACGTGCCTTGGAATTCTAGCTGGTCCAATAGAAACCTGTCTGAGATCTGGGACAACATGACATGGCTGCAATGGGATA<br>AGGAAATCAGCAACTACACCCAGATCATCTACGGCCTGCTTGAAGAGAGCCAGAACCAGCAGGAGAAGAACGAACAGGATCT<br>GCTGGCCCTGGAT

Supplementary Table 7 (continued). Nucleotide sequences of the constructs used in this study

BG505 SOSIP-T33-31A	ATGAAAAGGGGGCTGTGCTGTGTGCTGCTGCTGTGCGGGGCTGTGTTTGTGTCACCCAGTCAGGAAATCCACGCCAGATTCCG<br>GAGAGGAGCTAGGGCAGAAAACCTGTGGGTGACAGTCTACTATGGCGTGCCTGTCTGGAAGGACGCCGAGACCACACTGTTTT<br>GCGCTTCCGATGCCAAGGCTTACGAAACTAAGAAACACAATGTGTGGGCTACCCATTGCTGTGTCCCAACAGACCCAAACCCC<br>CAGGAAATCCACCTGGAGAATGTGACCGAGGAATTCAACATGTGGAAGAACAATATGGTGGAGCAGATGCATACAGACATCA<br>TTTCCCTGTGGGATCAGTCTCTGAAGCCTTGCGTGAAACTGACCCCACTGTGCGTCACACTCCAGTGTACAAACGTGACTAACA<br>ATATCACCGACGATATGCGCGGAGAACTGAAGAATTGTTCTTTCAACATGACTACCGAGCTGAGGGACAAGAAACAGAAAGT<br>GTACAGTCTGTTTTATCGCCTGGATGTGGTCCAGATCAATGAAAACCAGGGGAATAGAAGTAACAATTCAAACAAGGAGTACA<br>GGCTGATCAATTGCAACACCAGTGCCATTACACAGGCTTGTCCAAAAGTGTCATTTGAACCTATCCCAATTCATTATTGCGCAC<br>CTGCCGGCTTCGCCATCCTGAAGTGTAAAGATAAGAAGTTCAACGGCACTGGGCCCTGCACTAATGTGAGCACTGTCCAGTGT<br>ACCCACGGGATTAAGCCTGTGGTCTCCACCCAGCTGCTGCTGAATGGATCTCTGGCCGAGGAAGAAGTGATCATCCGGTCTGA<br>GAACATCACTAACAACGCTAAGAACATCCTGGTGCAGTTAAACGAATCCGTCCAGATTAATTGCACTAGACCTAACAATAACA<br>CCGTGAAATCTATCCGCATTGGACCCGGCCAGTGGTTTTATTATACCGGCGACATCATTGGGGATATCCGGCAGGCACACTGTA<br>ATGTGAGCAAGGCTACATGGAACGAGACTCTGGGGAAGGTGGTCAAACAGCTGCGCAAACATTTCGGAAATAACACCATCAT<br>TCGATTTGCCAATAGCTCCGGCGGGGACCTGGAAGTGACAACTCACAGCTTCAACTGCGGAGGCGAGTTCTTTTACTGTAACA<br>CAAGTGGCCTGTTTAATTCAACTTGGATCAGCAACACCTCCGTGCAGGGCTCTAATTCTACCGGCTCTAACGATAGTATCACAC<br>TGCCATGCCGGATTAAGCAGATCATTAATATGTGGCAGAGAATCGGGCAGGCAATGTATGCCCCCCCTATCCAGGGAGTGATT<br>CGATGTGTCAGCAATATCACAGGCCTGATTCTGACTAGAGACGGGGGATCAACAAACAGCACCACAGAGACTTTCAGGCCCGG<br>CGGGGGAGACATGCGAGATAACTGGCGGTCCGAACTGTACAAGTATAAAGTGGTCAAGATCGAGCCACTGGGAGTGGCACCA<br>ACCCGATGCAAAAGGCGAGTGGTCGGACGACGAAGAAGGCGACGAGCTGTGGGGATTGGAGCAGTCTCCCTGGGCTTTCTGG<br>GGGCCGCTGGATCTACAATGGGCGCAGCCAGTATGACTCTGACCGTCCAGGCCAGGAATCTGCTGTCAGGGATCGTGCAGCAG<br>CAGAGCAACCTGCTGCGCGCTCCTGAATGCCAGCAGCATCTGCTGAAGGACACCCACTGGGGCATCAAGCAGCTCCAGGCACG<br>AGTGCTGGCAGTCGAGCACTACCTGAGAGATCAGCAGCTGCTGGGAATCTGGGGGTGCAGCGGAAAGCTGATTTGCTGTACCA<br>ATGTGCCTTGGAACTCTAGTTGGAGCAATAGAAACCTGTCCGAAATCTGGGACAATATGACATGGCTCCAGTGGGATAAGGAG<br>ATTAGCAACTACACTCAGATCATCTACGGCCTGCTGGAAGAGTCCCAGAATCAGCAGGAGAAGAACGAGCAGGACCTGCTGG<br>AGCTGGACGGATCCGGTAGTGGATCAGGGTCTGGTGGGGAGGAAGTGGTCCTGATCACTGTGCCCAGTGCCCTGGTGGCTGTC<br>AAGATTGCTCATGCACTGGTGGAGGAAAGGCTGGCCGCTTGCGTGAACATCGTCCCTGGACTGACCAGTATCTACCGCGAGGA<br>AGGCAGCGTGGTGTCCGACCATGAGCTGCTGCTGCTGGTGAAGACCACAACTGATGCCTTTCCTAAGCTGAAAGAGCGAGTGA<br>AAGAACTGCACCCCTATGAGGTGCCCGAAATCGTCGCACTGCCAATTGCCGAGGGCAACCGGGAATACCTGGATTGGCTGAGA<br>GAGAATACAGGGCTGGAGTGA
BG505 SOSIP-T33-31B	ATGAAAAGGGGGCTGTGCTGTGTGCTGCTGCTGTGCGGGGCTGTGTTTGTGTCACCCAGTCAGGAAATCCACGCCAGATTCCG<br>GAGAGGAGCTAGGGCAGAAAACCTGTGGGTGACAGTCTACTATGGCGTGCCTGTCTGGAAGGACGCCGAGACCACACTGTTTT<br>GCGCTTCCGATGCCAAGGCTTACGAAACTAAGAAACACAATGTGTGGGCTACCCATTGCTGTGTCCCAACAGACCCAAACCCC<br>CAGGAAATCCACCTGGAGAATGTGACCGAGGAATTCAACATGTGGAAGAACAATATGGTGGAGCAGATGCATACAGACATCA<br>TTTCCCTGTGGGATCAGTCTCTGAAGCCTTGCGTGAAACTGACCCCACTGTGCGTCACACTCCAGTGTACAAACGTGACTAACA<br>ATATCACCGACGATATGCGCGGAGAACTGAAGAATTGTTCTTTCAACATGACTACCGAGCTGAGGGACAAGAAACAGAAAGT<br>GTACAGTCTGTTTTATCGCCTGGATGTGGTCCAGATCAATGAAAACCAGGGGAATAGAAGTAACAATTCAAACAAGGAGTACA<br>GGCTGATCAATTGCAACACCAGTGCCATTACACAGGCTTGTCCAAAAGTGTCATTTGAACCTATCCCAATTCATTATTGCGCAC<br>CTGCCGGCTTCGCCATCCTGAAGTGTAAAGATAAGAAGTTCAACGGCACTGGGCCCTGCACTAATGTGAGCACTGTCCAGTGT<br>ACCCACGGGATTAAGCCTGTGGTCTCCACCCAGCTGCTGCTGAATGGATCTCTGGCCGAGGAAGAAGTGATCATCCGGTCTGA<br>GAACATCACTAACAACGCTAAGAACATCCTGGTGCAGTTAAACGAATCCGTCCAGATTAATTGCACTAGACCTAACAATAACA<br>CCGTGAAATCTATCCGCATTGGACCCGGCCAGTGGTTTTATTATACCGGCGACATCATTGGGGATATCCGGCAGGCACACTGTA<br>ATGTGAGCAAGGCTACATGGAACGAGACTCTGGGGAAGGTGGTCAAACAGCTGCGCAAACATTTCGGAAATAACACCATCAT<br>TCGATTTGCCAATAGCTCCGGCGGGGACCTGGAAGTGACAACTCACAGCTTCAACTGCGGAGGCGAGTTCTTTTACTGTAACA<br>CAAGTGGCCTGTTTAATTCAACTTGGATCAGCAACACCTCCGTGCAGGGCTCTAATTCTACCGGCTCTAACGATAGTATCACAC<br>TGCCATGCCGGATTAAGCAGATCATTAATATGTGGCAGAGAATCGGGCAGGCAATGTATGCCCCCCCTATCCAGGGAGTGATT<br>CGATGTGTCAGCAATATCACAGGCCTGATTCTGACTAGAGACGGGGGATCAACAAACAGCACCACAGAGACTTTCAGGCCCGG<br>CGGGGGAGACATGCGAGATAACTGGCGGTCCGAACTGTACAAGTATAAAGTGGTCAAGATCGAGCCACTGGGAGTGGCACCA<br>ACCCGATGCAAAAGGCGAGTGGTCGGACGACGAAGAAGGCGACGAGCTGTGGGGATTGGAGCAGTCTCCCTGGGCTTTCTGG<br>GGGCCGCTGGATCTACAATGGGCGCAGCCAGTATGACTCTGACCGTCCAGGCCAGGAATCTGCTGTCAGGGATCGTGCAGCAG<br>CAGAGCAACCTGCTGCGCGCTCCTGAATGCCAGCAGCATCTGCTGAAGGACACCCACTGGGGCATCAAGCAGCTCCAGGCACG<br>AGTGCTGGCAGTCGAGCACTACCTGAGAGATCAGCAGCTGCTGGGAATCTGGGGGTGCAGCGGAAAGCTGATTTGCTGTACCA<br>ATGTGCCTTGGAACTCTAGTTGGAGCAATAGAAACCTGTCCGAAATCTGGGACAATATGACATGGCTCCAGTGGGATAAGGAG<br>ATTAGCAACTACACTCAGATCATCTACGGCCTGCTGGAAGAGTCCCAGAATCAGCAGGAGAAGAACGAGCAGGACCTGCTGG<br>AGCTGGACGGATCCGGATCTGGATCTGGATCTGGATCTGTGCGCGGCATCCGGGGAGCTATCACCGTGGAGGAGGACACACCA<br>GCTGCTATCCTGGCTGCTACCATCGAGCTGCTGCTGAAGATGCTGGAGGCCAACGGCATCCAGAGCTACGAGGAGCTGGCCGC<br>TGTGATCTTCACCGTGACAGAGGATCTGACATCCGCCTTTCCAGCTGAGGCTGCTAGGCTGATCGGAATGCACCGGGTGCCCCT<br>GCTGTCCGCCCGCGAGGTGCCCGTGCCTGGCTCTCTGCCCAGAGTGATCAGAGTGCTGGCTCTGTGGAACACCGACACACCAC<br>AGGATAGAGTGCGCCACGTGTATCTGAATGAGGCCGTGCGGCTGAGGCCAGATCTGGAGTCTGCTCAGCTGGAG

Supplementary Table 8. EMDB and PDB accession IDs for the models and maps produced in this study

Sample description	EMDB ID	PDB ID
nsEM maps of Grp 1 polyclonal samples (Wk 26)	23175	N/A
nsEM maps of Grp 2 polyclonal samples (Wk 26)	23176	N/A
nsEM maps of Grp 3 polyclonal samples (Wk 8)	23177	N/A
nsEM maps ofGrp 3 polyclonal samples (Wk 10)	23178	N/A
nsEM maps of Grp 3 polyclonal samples (Wk 26)	23179	N/A
nsEM maps of Grp 3 polyclonal samples (Wk 38)	23180	N/A
nsEM map of Rh.33172 (Wk 26) polyclonal sample (antigen: BG505 SOSIP.v3)	23182	N/A
nsEM map of Rh.33172 (Wk 26) polyclonal sample (antigen: BG505 SOSIP.v5.2(7S) N241/N289)	23181	N/A
nsEM map of Rh.33311 (Wk 26) polyclonal sample (antigen: BG505 SOSIP.v3)	23183	N/A
nsEM map of Rh.33311 (Wk 26) polyclonal sample (antigen: BG505 SOSIP.v5.2(7S) N241/N289)	23184	N/A
nsEM map of the BG505 SOSIP-T33-31A nanoparticle component	23185	N/A
nsEM map of the BG505 SOSIP-T33-31A nanoparticle component	23186	N/A
cryoEM map and model of the BG505 SOSIP (A) subparticle of the BG505 SOSIP-T33-31 nanoparticle	23218	7L7T
cryoEM map of the BG505 SOSIP (B) subparticle of the BG505 SOSIP-T33-31 nanoparticle	23219	7L7U
cryoEM map and model of the T33-31 core subparticle of the BG505 SOSIP-T33-31 nanoparticle	23222	7L85
cryoEM map and model of the Rh.32034 pAbC-1 (antigen: BG505 SOSIP MD39)	23223	7L86
cryoEM map and model of the Rh.32034 pAbC-2 (antigen: BG505 SOSIP MD39)	23224	7L87
cryoEM map and model of the Rh.32034 pAbC-3 (antigen: BG505 SOSIP MD39)	23225	7L88
cryoEM map and model of the Rh.32034 pAbC-4 (antigen: BG505 SOSIP MD39)	23226	7L89
cryoEM map and model of the Rh.33104 pAbC-1 (antigen: BG505 SOSIP MD39)	23227	7L8A
cryoEM map and model of the Rh.33104 pAbC-2 (antigen: BG505 SOSIP MD39)	23228	7L8B
cryoEM map and model of the Rh.33104 pAbC-3 (antigen: BG505 SOSIP MD39)	23229	7L8C
cryoEM map and model of the Rh.33104 pAbC-4 (antigen: BG505 SOSIP MD39)	23230	7L8D
cryoEM map and model of the Rh.33311 pAbC-1 (antigen: BG505 SOSIP.v5.2 N241/N289)	23236	7L8T
cryoEM map and model of the Rh.33311 pAbC-2 (antigen: BG505 SOSIP.v5.2 N241/N289)	23237	7L8U
cryoEM map and model of the Rh.33311 pAbC-3 (antigen: BG505 SOSIP.v5.2 N241/N289)	23238	7L8W
cryoEM map and model of the Rh.33311 pAbC-4 (antigen: BG505 SOSIP.v5.2 N241/N289)	23239	7L8X
cryoEM map and model of the Rh.33311 pAbC-5 (antigen: BG505 SOSIP.v5.2 N241/N289)	23240	7L8Y
cryoEM map of the Rh.33311 pAbC-6 (antigen: BG505 SOSIP.v5.2 N241/N289)	23241	N/A
cryoEM map and model of the Rh.33311 pAbC-7 (antigen: BG505 SOSIP.v5.2 N241/N289)	23242	7L8Z
cryoEM map and model of the Rh.33311 pAbC-8 (antigen: BG505 SOSIP.v5.2 N241/N289)	23243	7L90
cryoEM map and model of the Rh.33172 pAbC-1 (antigen: BG505 SOSIP.v5.2(7S) N241/N289)	23231	7L8E
cryoEM map and model of the Rh.33172 pAbC-2 (antigen: BG505 SOSIP.v5.2(7S) N241/N289)	23232	7L8F
cryoEM map and model of the Rh.33172 pAbC-3 (antigen: BG505 SOSIP.v5.2(7S) N241/N289)	23233	7L8G
cryoEM map and model of the Rh.33172 pAbC-4 (antigen: BG505 SOSIP.v5.2(7S) N241/N289)	23235	7L8S
cryoEM map of the Rh.33172 pAbC-5 (antigen: BG505 SOSIP.v5.2(7S) N241/N289)	23234	N/A

A

	A501C	A605C	I559P	E64K	A316W	A73C	A561C	S613T	P240T	S241N	T290E	P291S	F288L	M271I	T106E	Q658K	R304V	A319Y	N363Q	F519S	A561P	L568D	V570H	R585H
BG505 SOSIP.v3	green	green	green	red	red	red	red	red	red	red	red	red	red	red	red	red	red	red	red	red	red	red	red	red
BG505 SOSIP.v5.2 N241/N289	green	green	green	green	green	green	green	green	green	green	green	green	green	green	red	red	red	red	red	red	red	red	red	red
BG505 SOSIP MD39	green	green	green	red	red	red	red	red	red	red	red	red	green	green	green	green	green	green	green	green	green	green	green	green
BG505 SOSIP.v5.2(7S) N241/N289	green	green	green	green	green	green	green	red	green	green	green	green	green	green	red	red	green	green	red	green	red	green	green	green

B

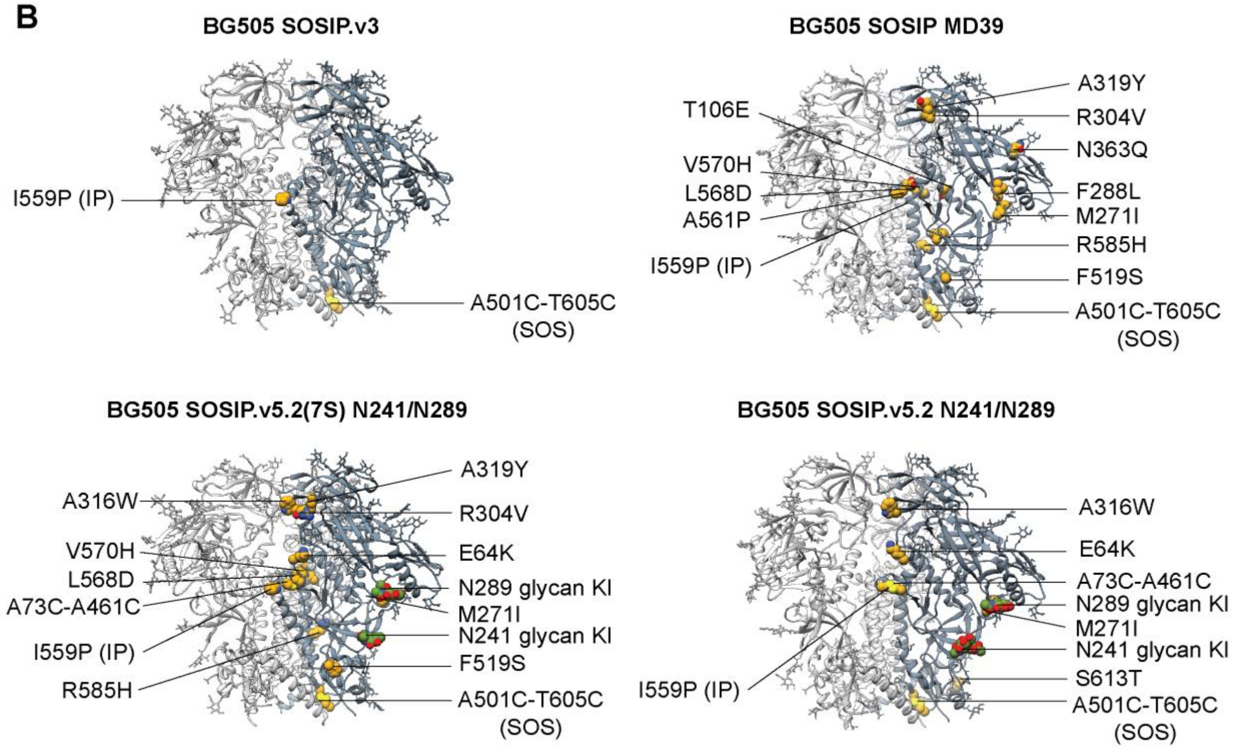

Supplementary Figure 1. Stabilizing and glycan knock-in mutations in BG505 SOSIP constructs used in this study. In panel [A] the mutations are presented in a table format (green – mutation present; red – mutation not present), and in panel [B] they are displayed on a structure of BG505 SOSIP. For simplicity the mutations are shown for one protomer only (dark-gray ribbon). BG505 SOSIP is displayed using ribbon representation and sphere representation was used for mutated residues (yellow – stabilizing mutations; green – glycan knock-ins).

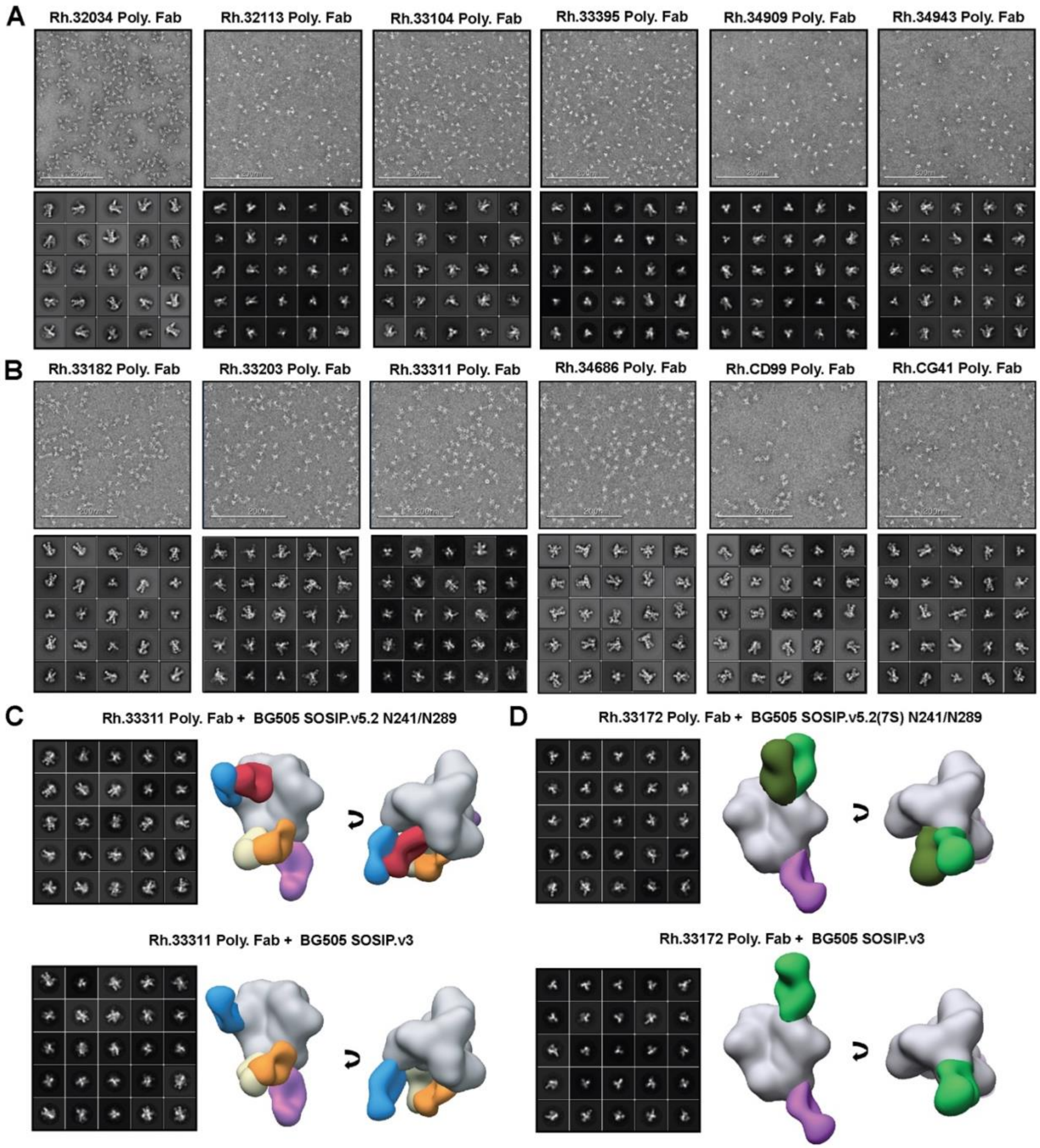

Supplementary Figure 2. Extended nsEMPEM data. [A,B] Representative raw EM micrographs (top) and 2D class averages (bottom) from the nsEMPEM datasets used for the generation of Grp 1 and Grp 2 composite figures presented in Figure 1d. Immune complexes were generated with BG505 SOSIP MD39 for Grp 1 and BG505 SOSIP.v5.2 N241/N289 for Grp 2. **[C]** An equivalent amount of polyclonal Fab sample from animal Rh.33311 (wk 26) was complexed with BG505 SOSIP.v5.2 N241/N289 and BG505 SOSIP.v3 and imaged using EM to investigate the effect engineered stabilizing mutations have on antibody binding. Raw EM micrographs, 2D class averages and composite figures for the two nsEMPEM experiments are shown. **[D]** Similar experiment as in panel **[C]** was performed with Rh.33172 polyclonal Fab (wk 26). BG505 SOSIP.v5.2(7S) N241/N289 and BG505 SOSIP.v3 antigens were used for complexing. For color scheme, see the legend in Figure 1. In panel **[D]** two different shades of green (olive and bright green) were used to depict different polyclonal antibody classes targeting partially overlapping V1/V2/V3 epitopes. The scale bar in raw micrographs is 200 nm and the box size for 2D classes is 39.4 nm (192 pixels). The nsEMPEM experiments have been performed once.

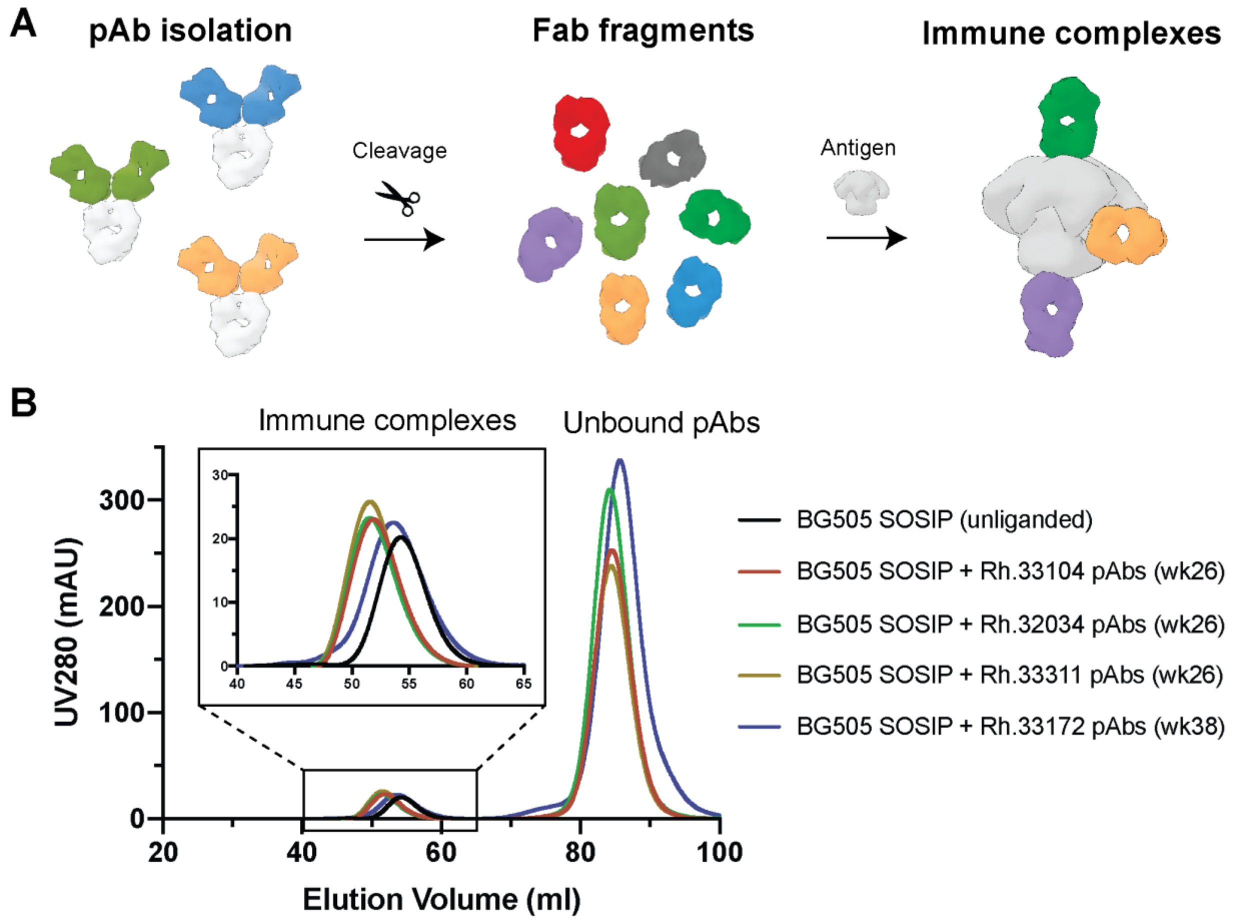

Supplementary Figure 3. Assembly and SEC purification of the polyclonal immune complexes. [A] Schematic overview of the early steps of cryoEMPEM workflow leading to assembly of polyclonal immune complexes. **[B]** SEC purification of polyclonal immune complexes. The details of the experiment are provided in the Methods section. SEC chromatograms of individual samples are depicted in different colors. Unliganded BG505 SOSIP curve is displayed for reference. Note the shift in elution volume of immune complex peaks compared to the BG505 SOSIP reference, indicating the increase in size of the assembled immune complexes. All fractions corresponding to the immune complex peak (~45-60 mL) were pooled, concentrated and used for preparation of cryoEM grids.

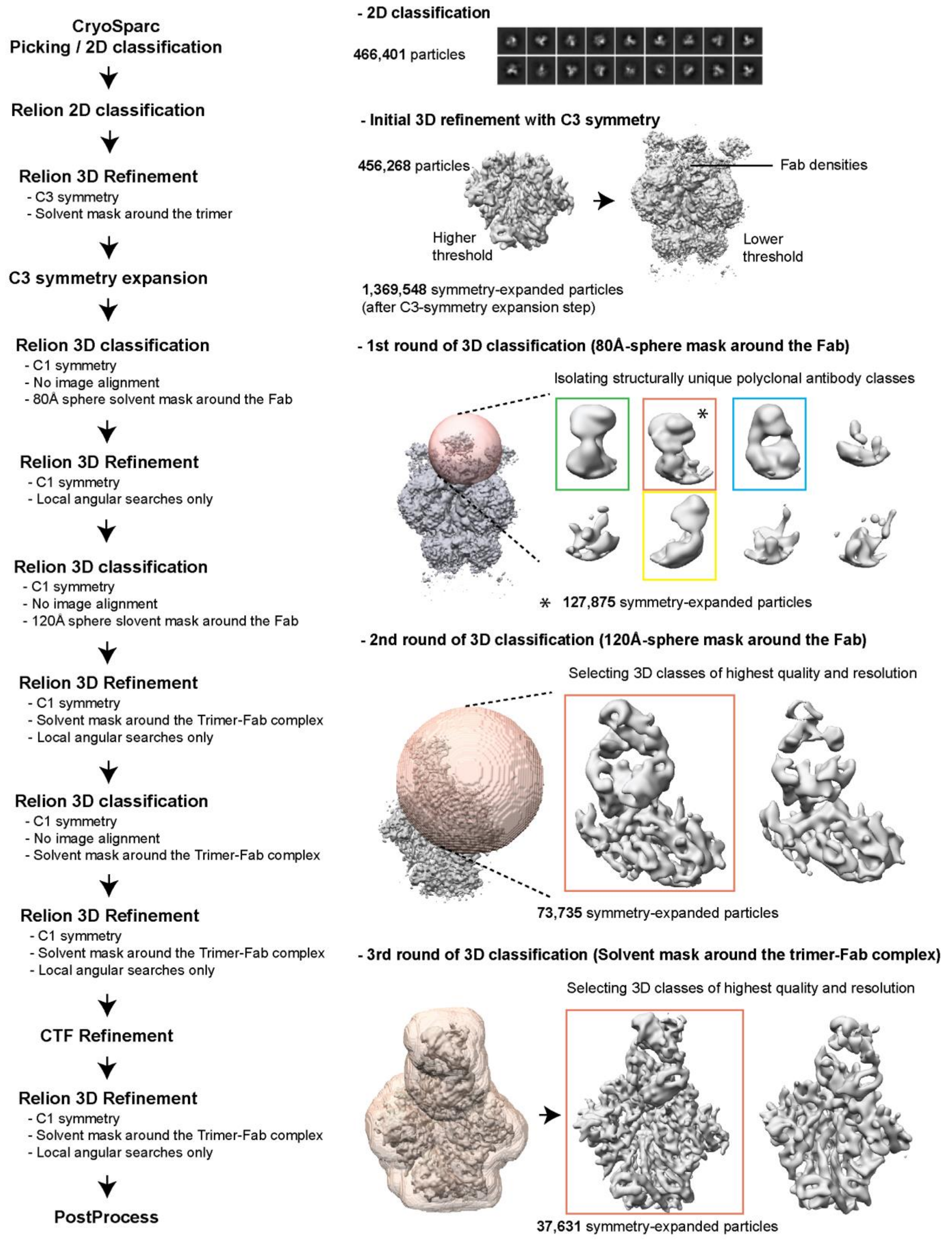

Supplementary Figure 4. Schematic representation of the focused classification approach used for processing of cryoEMPEM data. Full data processing workflow is shown on the left and the examples of intermediate results from the Rh.33172 dataset are shown on the right.

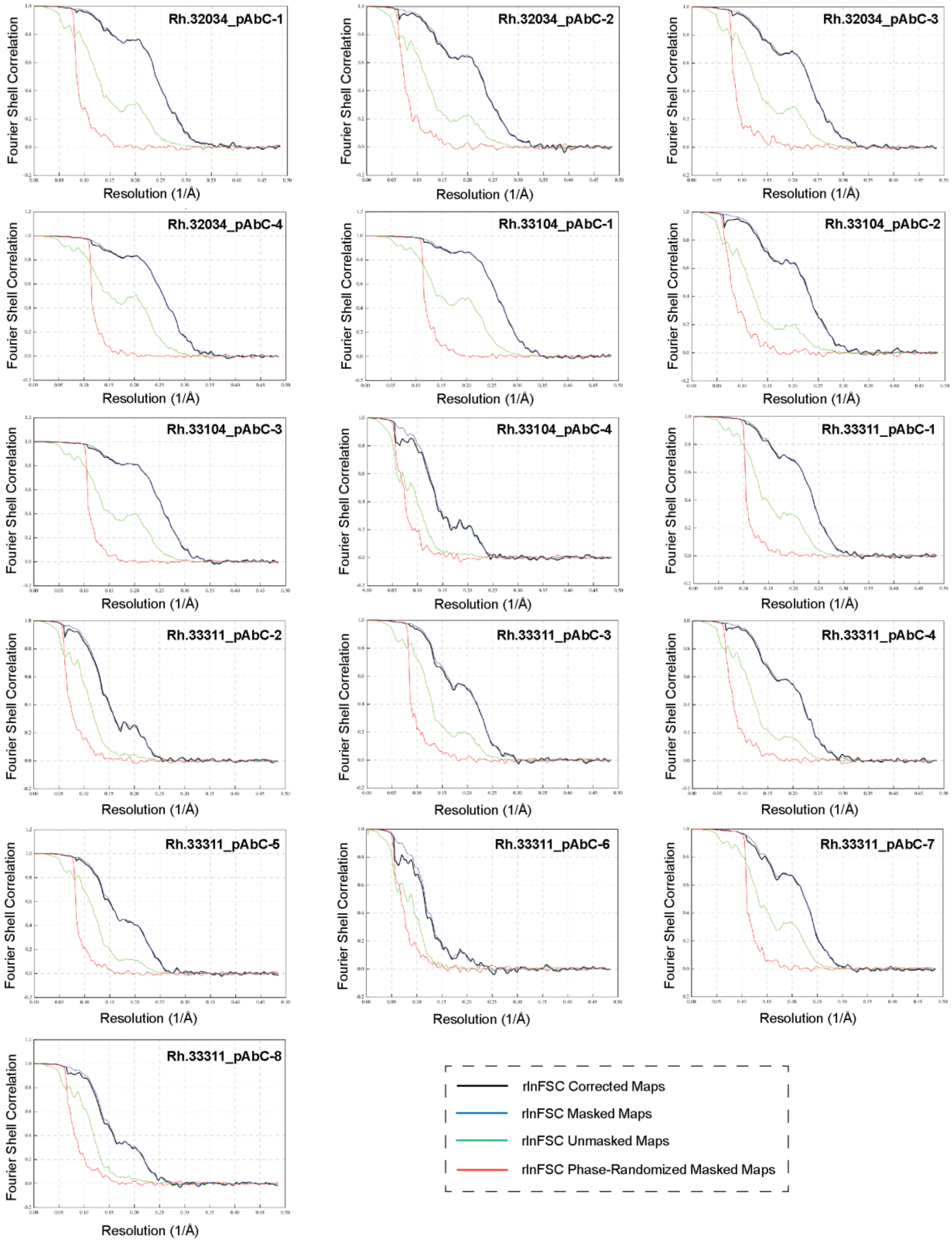

Supplementary Figure 5. FSC resolution plots for EM maps reconstructed by cryoEMPEM analysis of polyclonal Fab samples isolated from animals Rh.32034, Rh.33104 and Rh.33311. The plots were generated in Relion/3.0.

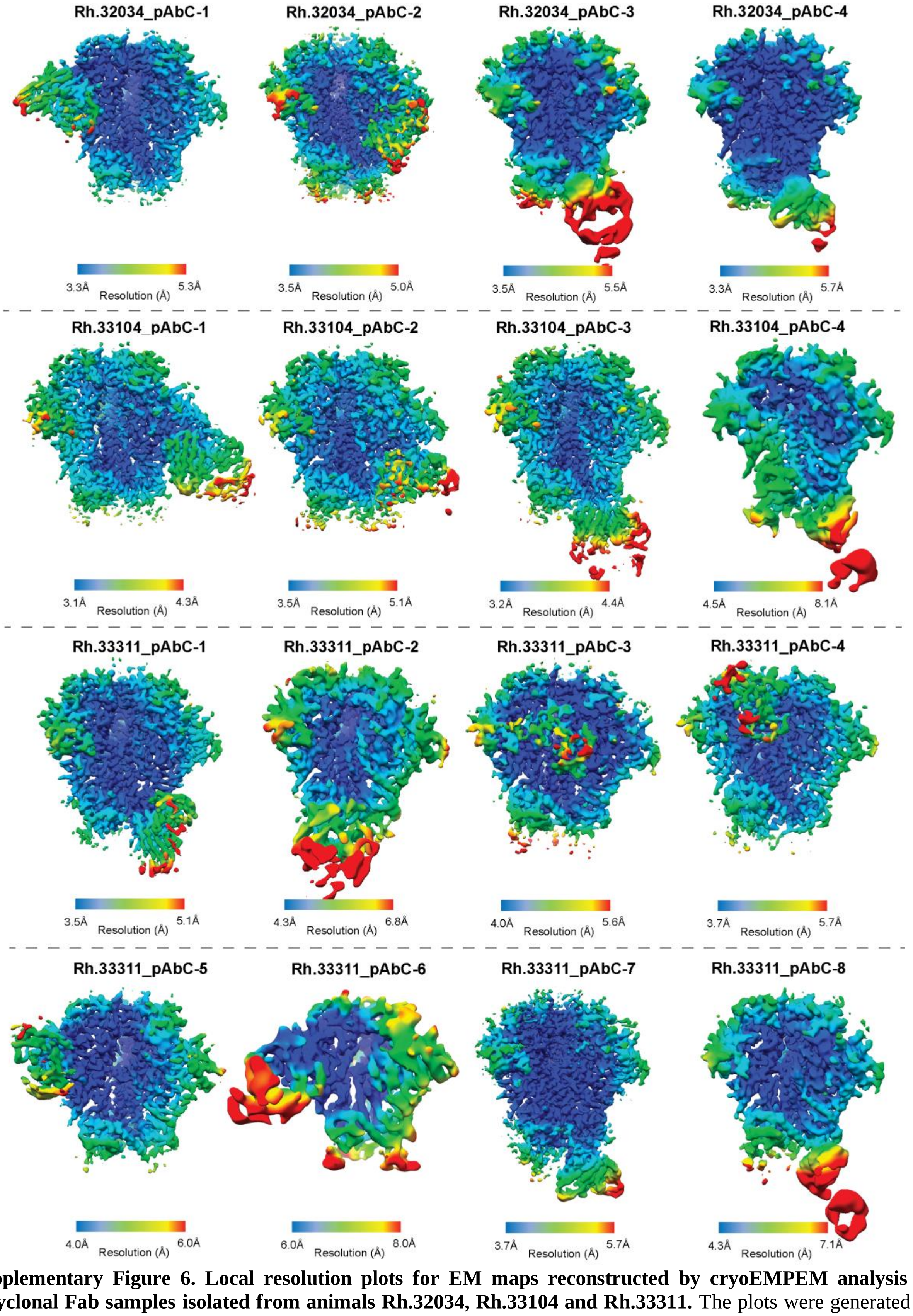

Supplementary Figure 6. Local resolution plots for EM maps reconstructed by cryoEMPEM analysis of polyclonal Fab samples isolated from animals Rh.32034, Rh.33104 and Rh.33311. The plots were generated in Relion/3.0.

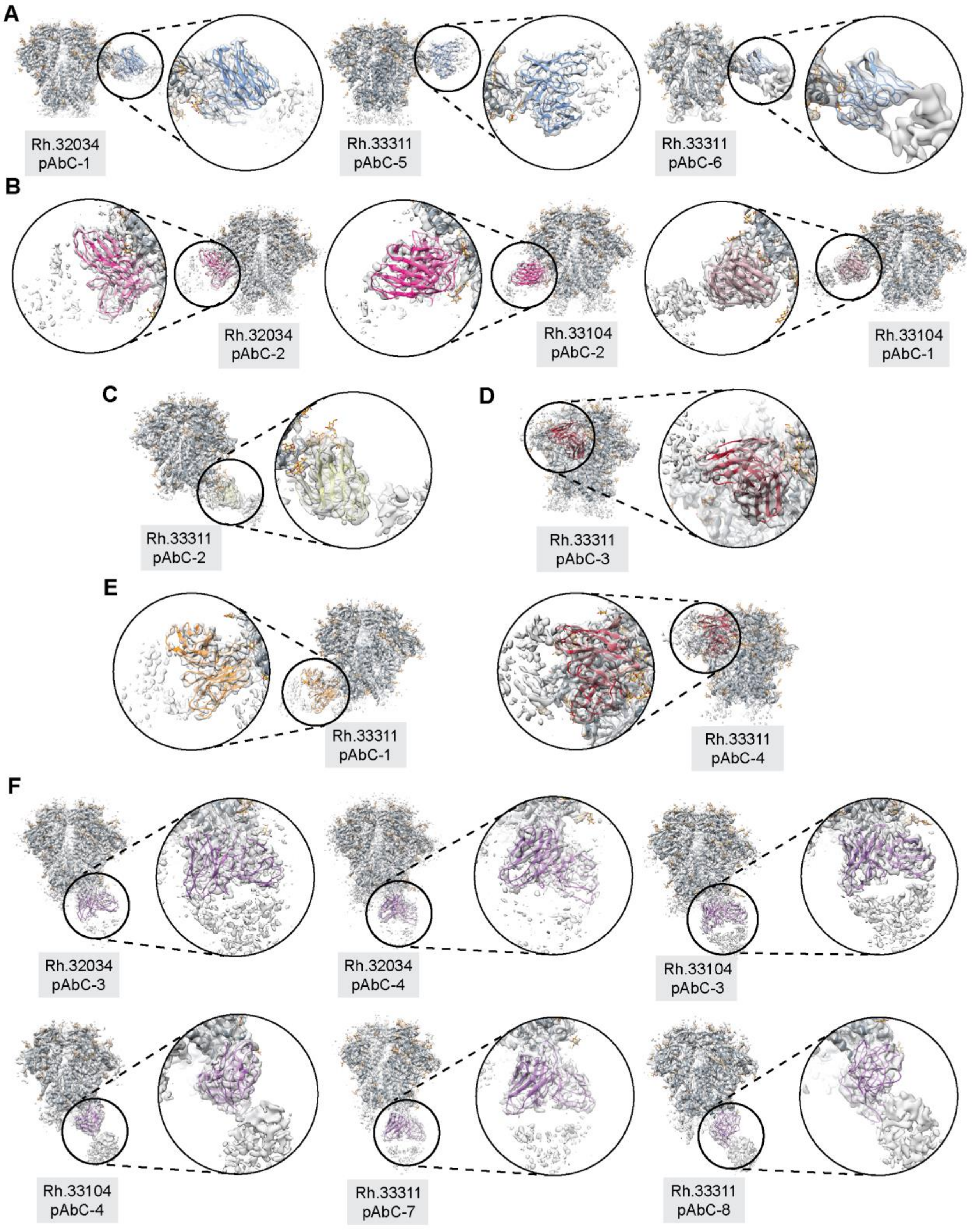

Supplementary Figure 7. Models-to-map fit of BG505 SOSIP antigens in complex with reconstructed polyclonal antibodies. C3/V5 epitope **[A]**, N241/N289 glycan hole **[B]**, N611 glycan epitope **[C]**, gp120-gp120 interface **[D]**, fusion peptide **[E]**, and base of the trimer **[F]**. A close-up view of the antibody backbone model (poly-Alanine) in the corresponding density is shown.

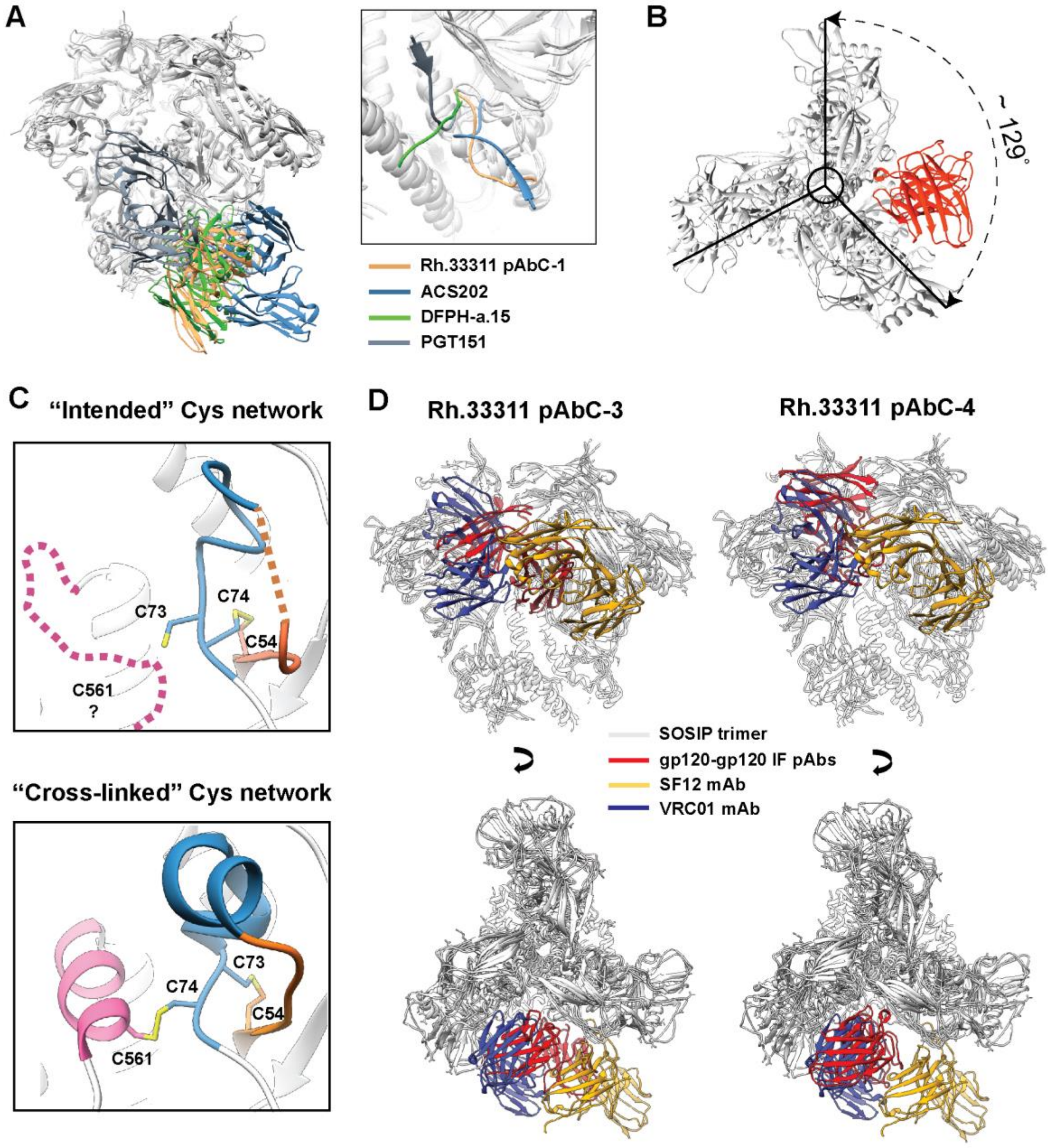

Supplementary Figure 8. Supporting structural data associated with Figures 3 and 4. [A] The alignment of structures of FP-targeting bNAbs and Rh.33311 pAbC-1. The figure on the left shows full Fab complex structures. FP conformation in the antibody-bound state is shown in the panel on the right. **[B]** A view down the 3-fold symmetry axis of the BG505 SOSIP trimer in the Rh.33311 pAbC-4 complex structure showing the splaying of the trimer upon antibody binding. **[C]** The differences in architecture of the C1 loop (residues 53-60 depicted in orange; residues 61-75 depicted in blue) and the HR1 region (residues 559-567 in pink), observed between the BG505 SOSIP.v5.2 structure with "intended" pairing of cysteines (PDB ID: 6VO0) and BG505 SOSIP.v5.2 model from Rh.33311 pAbC-3 ("cross-linked" Cys network). Note that altered Cys network and the binding of Rh.33311 pAbC-3 polyclonal antibody to the C1 loop stabilize the entire region and allow to build atomic coordinates for the underlying C1 and HR1 residues. **[D]** Overlay of the Rh.33311 pAbC-3 (left) and pAbC-4 (right) structures with monoclonal antibodies VRC01 (PDB ID: 6V8X) and SF12 (PDB ID: 6OKP), targeting the CD4bs and the silent face epitopes, respectively. Note the overlapping footprint between the gp120-gp120 interface-targeting pAbs and the two mAbs.

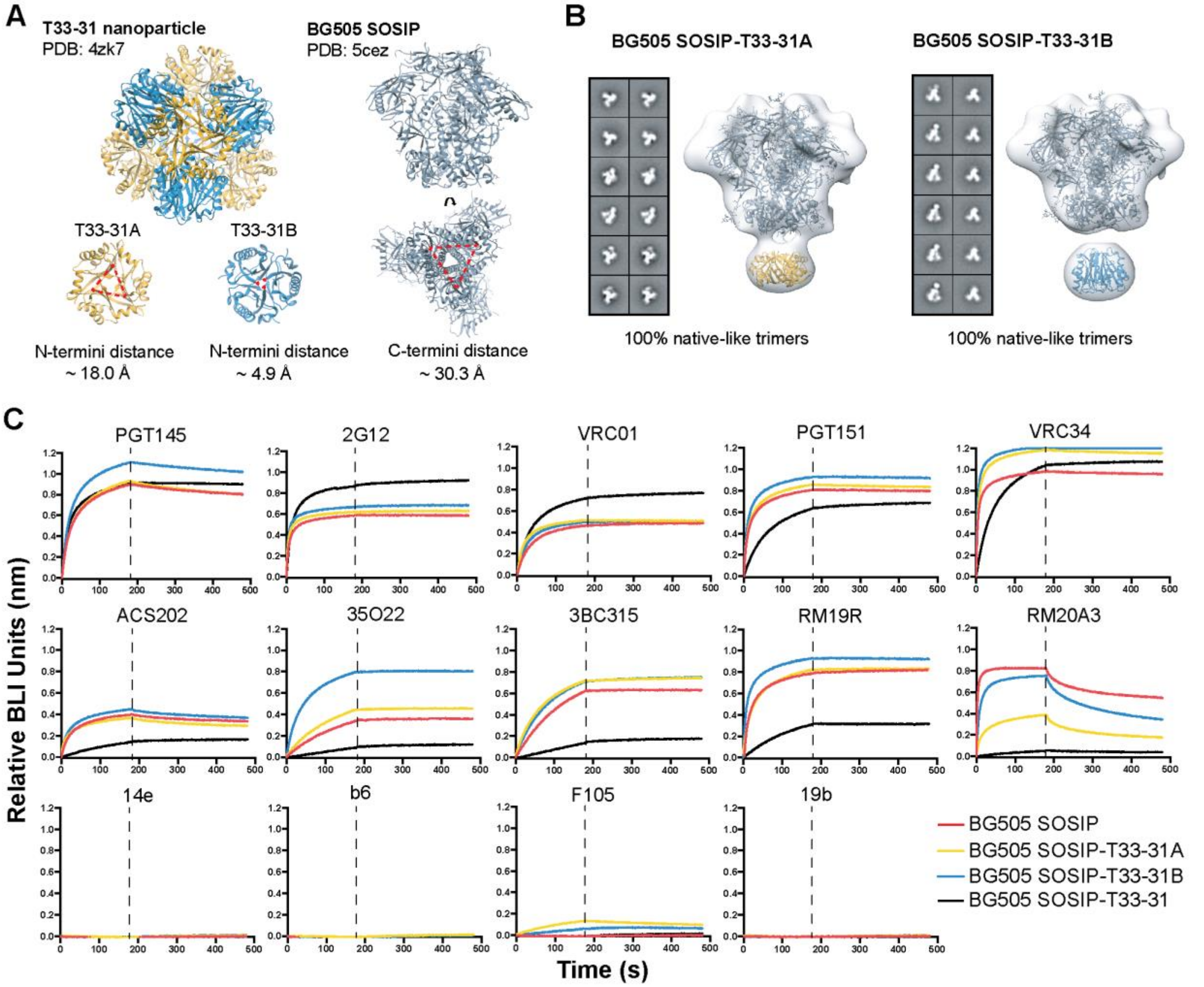

Supplementary Figure 9. Design, structural and antigenic evaluation of the BG505 SOSIP-T33-31 nanoparticle immunogen. **[A]** The structure of the T33-31 two-component nanoparticle is shown in the top left corner and the BG505 SOSIP structure is shown on the right (ribbon representation is used; component A is colored yellow; component B is colored blue; BG505 SOSIP is colored gray). The distances between corresponding fusion residues in each trimeric building block (N-termini for T33-31A and T33-31B and C-termini for BG505 SOSIP) are shown in the bottom panels and these residues are connected by red dashed lines. **[B]** nsEM characterization of the two nanoparticle components fused to C-termini of BG505 SOSIP trimers. 2D-class averages are shown on the left in each panel; reconstructed EM maps are displayed on the right as transparent white mesh with BG505 SOSIP and nanoparticle components docked into the corresponding parts of the map. **[C]** BLI-based antigenicity analysis of BG505-SOSIP.v5.2(7S) N241/N289 antigens as free trimers, fused to T33-31A and T33-31B nanoparticle building blocks and assembled into a T33-31 nanoparticle. Antibody name is indicated above the corresponding panel.

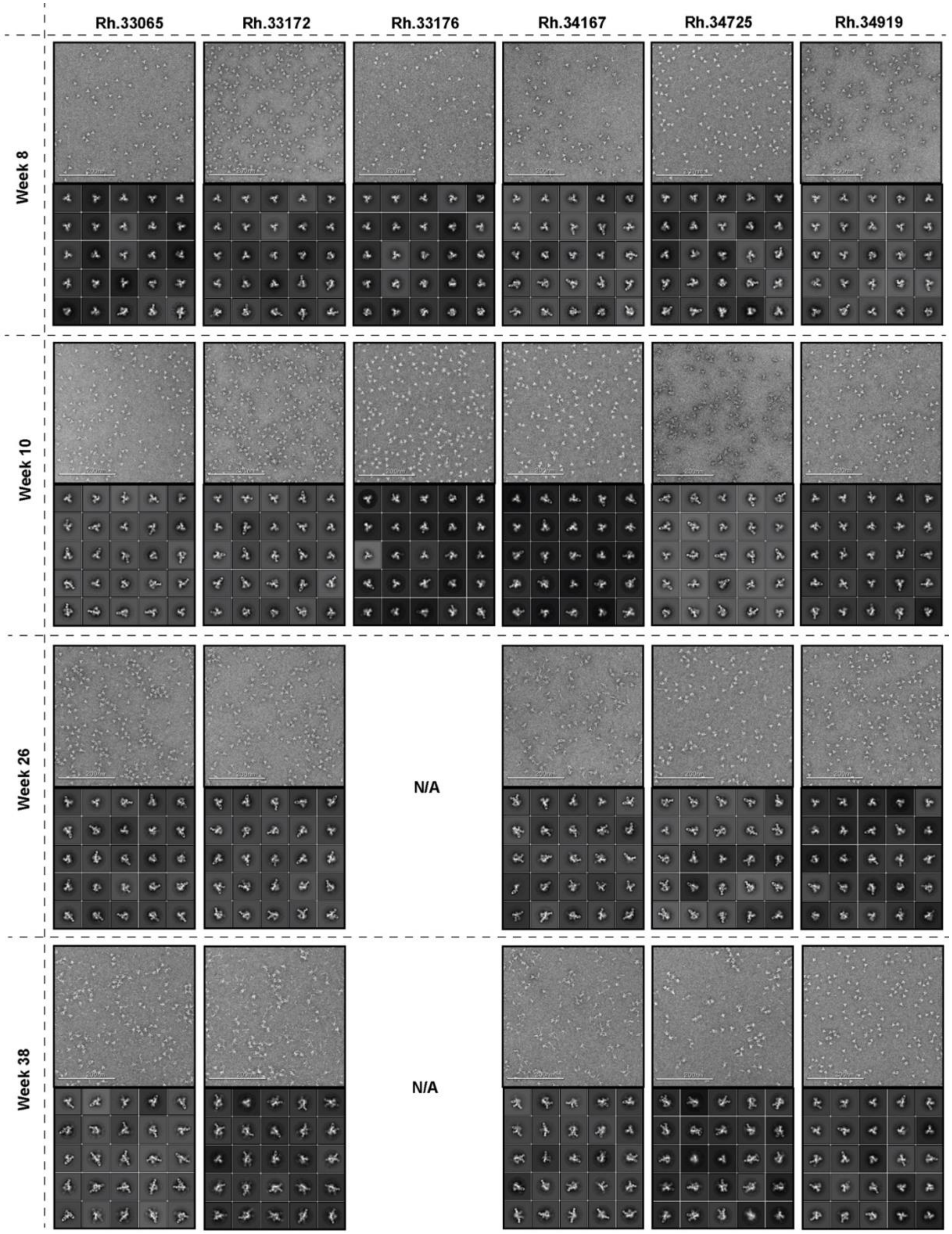

Supplementary Figure 10. Extended nsEMPEM data. Representative raw EM micrographs (top) and 2D class averages (bottom) from the nsEMPEM datasets used for the generation of Grp 3 composite figures presented in Figure 6d. Animal ID is shown on top and the corresponding time points at which plasma samples were extracted are shown on the left. The scale bar in raw micrographs is 200 nm and the box size for 2D classes is 39.4 nm (192 pixels). The nsEMPEM experiments have been performed once.

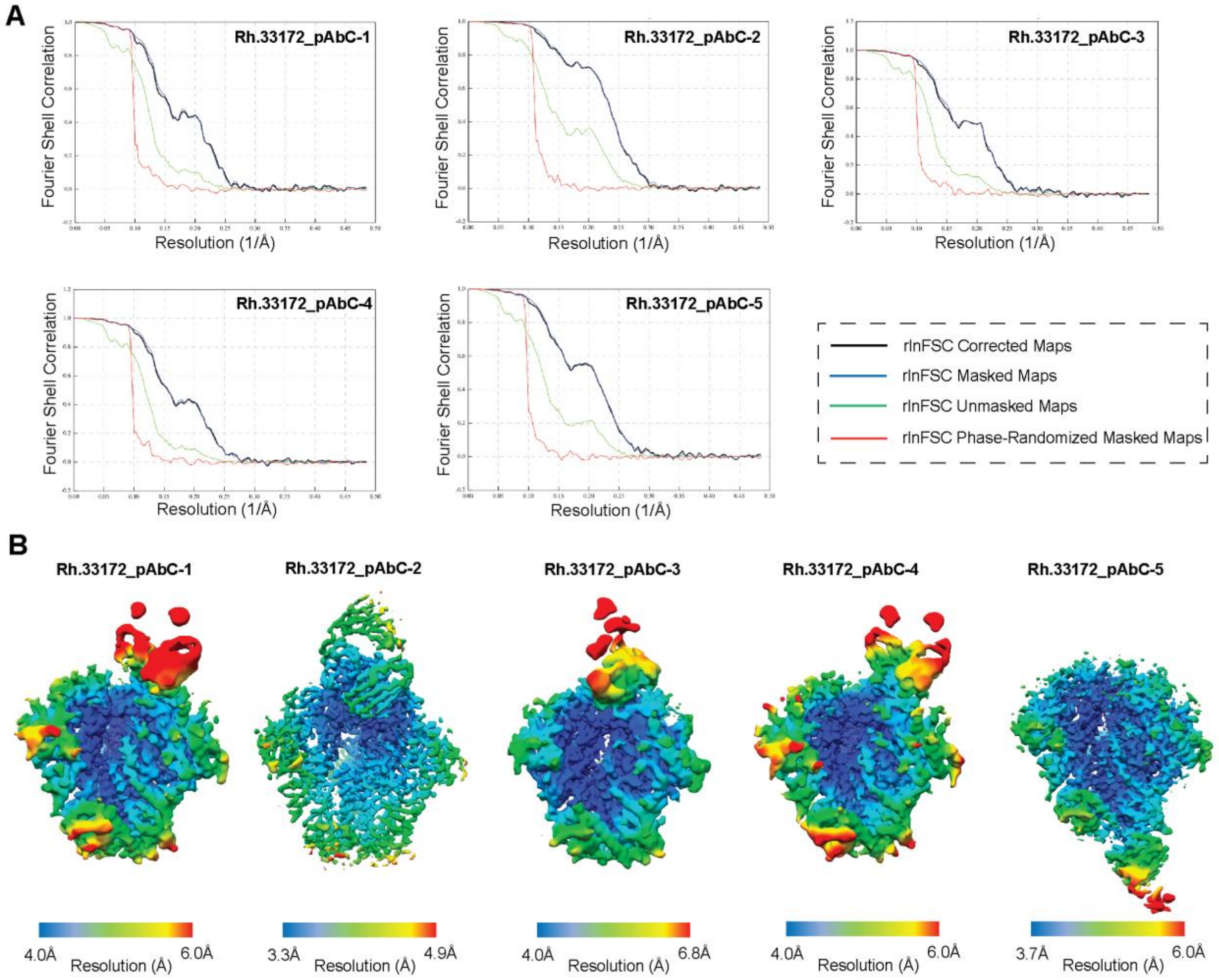

Supplementary Figure 11. Extended cryoEMPEM data associated with Figure 7. FSC resolution plots **[A]**, and local resolution plots **[B]** for EM maps reconstructed by cryoEMPEM analysis of polyclonal Fab sample isolated from animal Rh.33172. The plots were generated in Relion/3.0.
