# Supplementary material for: Polyclonal antibody responses to HIV Env immunogens resolved using cryoEM
Source: Nat Commun. 2021 Aug 10;12:4817. doi: 10.1038/s41467-021-25087-4 (PMC8355326; doi:10.1038/s41467-021-25087-4)
Supplement: Supplementary file 3 — Reporting Summary [file 41467_2021_25087_MOESM3_ESM.pdf]

nature research

Corresponding author(s): Andrew B. Ward

Last updated by author(s): Jul 13, 2021

Reporting Summary

Nature Research wishes to improve the reproducibility of the work that we publish. This form provides structure for consistency and transparency in reporting. For further information on Nature Research policies, see our Editorial Policies and the Editorial Policy Checklist.

Statistics

For all statistical analyses, confirm that the following items are present in the figure legend, table legend, main text, or Methods section.

n/a	Confirmed	
☐	☒	The exact sample size (*n*) for each experimental group/condition, given as a discrete number and unit of measurement
☐	☒	A statement on whether measurements were taken from distinct samples or whether the same sample was measured repeatedly
☒	☐	The statistical test(s) used AND whether they are one- or two-sided<br>*Only common tests should be described solely by name; describe more complex techniques in the Methods section.*
☒	☐	A description of all covariates tested
☒	☐	A description of any assumptions or corrections, such as tests of normality and adjustment for multiple comparisons
☐	☒	A full description of the statistical parameters including central tendency (e.g. means) or other basic estimates (e.g. regression coefficient) AND variation (e.g. standard deviation) or associated estimates of uncertainty (e.g. confidence intervals)
☒	☐	For null hypothesis testing, the test statistic (e.g. *F*, *t*, *r*) with confidence intervals, effect sizes, degrees of freedom and *P* value noted<br>*Give P values as exact values whenever suitable.*
☒	☐	For Bayesian analysis, information on the choice of priors and Markov chain Monte Carlo settings
☒	☐	For hierarchical and complex designs, identification of the appropriate level for tests and full reporting of outcomes
☒	☐	Estimates of effect sizes (e.g. Cohen's *d*, Pearson's *r*), indicating how they were calculated

Our web collection on statistics for biologists contains articles on many of the points above.

Software and code

Policy information about availability of computer code

Data collection	Leginon (beta version)
Data analysis	Relion (v3.0), CryoSPARC (v2.15), Octet System Data Analysis (v9.0), Excel (v16.43), GraphPad Prism (v8.4.3), Appion (v1), UCSF Chimera (v1.13), MotionCor (v2), MolProbity (v4.2), EMRinger (version N/A), Localized Reconstruction (v1.2.0), GCTF (v1.06_sm_30_cu8.0_x86_64), Coot (v0.9-pre)

For manuscripts utilizing custom algorithms or software that are central to the research but not yet described in published literature, software must be made available to editors and reviewers. We strongly encourage code deposition in a community repository (e.g. GitHub). See the Nature Research guidelines for submitting code & software for further information.

Data

Policy information about availability of data

All manuscripts must include a data availability statement. This statement should provide the following information, where applicable:

- Accession codes, unique identifiers, or web links for publicly available datasets
- A list of figures that have associated raw data
- A description of any restrictions on data availability

3D maps and models from the EM analysis have been deposited to the Electron Microscopy Databank (http://www.emdatabank.org/) and the Protein Data Bank (http://www.rcsb.org/), respectively. EMDB IDs: 23175-23186, 23218-9, 23222-23243. PDB IDs: 7L7T, 7L7U,7L85, 7L86, 7L87, 7L88, 7L89, 7L8A, 7L8B,7L8C,7L8D,7L8T,7L8U, 7L8W, 7L8X,7L8Y, 7L8Z, 7L90, 7L8E, 7L8F, 7L8G, 7L8S.
In the work we have also used existing structures from the PDB database (http://www.rcsb.org/) with accession numbers: 6VFL, 4KTE, 4KTD, 4RFE, 4Q2Z, 4ZK7

Field-specific reporting

Please select the one below that is the best fit for your research. If you are not sure, read the appropriate sections before making your selection.

- [x] Life sciences
- [ ] Behavioural & social sciences
- [ ] Ecological, evolutionary & environmental sciences

For a reference copy of the document with all sections, see nature.com/documents/nr-reporting-summary-flat.pdf

Life sciences study design

All studies must disclose on these points even when the disclosure is negative.

Sample size	Sizes of animal groups were minimized for ethical concerns but still provide statistical significance. For ELISA and viral neutralization assays, the experiments were performed in triplicates (n=3) which is a field-accepted standard for these types of experiments.
Data exclusions	no exclusions
Replication	ELISA and neutralization experiments were repeated in triplicates (n=3). All EMPEM experiments were performed once (n=1) No Data was excluded.
Randomization	Animals were assigned to experimental groups randomly with respect to their age or gender. Randomization and experimental group assignments are not applicable to other experiments performed in this study.
Blinding	No investigators were blinded. This is an early, proof-of-concept immunization study without direct connection to any ongoing/planned clinical trials. Blinding was considered but it was estimated to be unnecessary.

Reporting for specific materials, systems and methods

We require information from authors about some types of materials, experimental systems and methods used in many studies. Here, indicate whether each material, system or method listed is relevant to your study. If you are not sure if a list item applies to your research, read the appropriate section before selecting a response.

Materials & experimental systems

n/a	Involved in the study	
☐	☒	Antibodies
☐	☒	Eukaryotic cell lines
☒	☐	Palaeontology and archaeology
☐	☒	Animals and other organisms
☒	☐	Human research participants
☒	☐	Clinical data
☒	☐	Dual use research of concern

Methods

n/a	Involved in the study	
☒	☐	ChIP-seq
☒	☐	Flow cytometry
☒	☐	MRI-based neuroimaging

Antibodies

Antibodies used	Antibodies were isolated and purified from animal sera samples. mAbs PGT145, 2G12, VRC01, PGT151, VRC34, ACS202, 35O22, 3BC315, RM19R, RM20A3, 14e, b6, F105, 19b, 12N were recombinantly expressed and purified as previously described and properly referenced. AP-conjugated AffiniPure goat anti-human IgG was used for ELISA experiments and was purchased from Jackson Immunoresearch (Cat # 109-055-097, Lot # 141947).
Validation	No novel antibodies were discovered or validated.

Eukaryotic cell lines

Policy information about cell lines

Cell line source(s)	FreeStyle293F (ThermoFisher Sci, Cat # A14528)
Authentication	No authentication
Mycoplasma contamination	Mycoplasma is tested on a monthly basis. All cell lines used are confirmed negative.
Commonly misidentified lines (See ICLAC register)	No commonly misidentified cell lines were used in the study

Animals and other organisms

Policy information about studies involving animals; ARRIVE guidelines recommended for reporting animal research

Laboratory animals	Macaca mulatta (female and male, age ranged from 3 to 8 years)
Wild animals	No wild animals were used in the study.
Field-collected samples	No field collected samples were used in the study.
Ethics oversight	Emory University Institutional Animal Care and Use Committee protocol 201700723

Note that full information on the approval of the study protocol must also be provided in the manuscript.
